# Modulating tumor infiltrating myeloid cells to enhance bispecific antibody-driven T cell infiltration and anti-tumor response

**DOI:** 10.1186/s13045-021-01156-5

**Published:** 2021-09-08

**Authors:** Jeong A. Park, Linlin Wang, Nai-Kong V. Cheung

**Affiliations:** Department of Pediatrics, Memorial Sloan Kettering Cancer Center, 1275 York Avenue, New York, NY 10065 USA

**Keywords:** Bispecific antibody, Dexamethasone, Disialogangliosides, Ex vivo bispecific antibody armed T-cells (EATs), Human epidermal growth factor receptor 2 (HER2), Immunotherapy, Myeloid-derived suppressor cell, T cell, Tumor infiltrating myeloid cell, Tumor microenvironment, Tumor-associated macrophage

## Abstract

**Background:**

Tumor microenvironment (TME) is a dynamic cellular milieu to promote tumor angiogenesis, growth, proliferation, and metastasis, while derailing the host anti-tumor response. TME impedes bispecific antibody (BsAb) or chimeric antigen receptor (CAR)-driven T cells infiltration, survival, and cytotoxic efficacy. Modulating tumor infiltrating myeloid cells (TIMs) could potentially improve the efficacy of BsAb.

**Methods:**

We evaluated the effects of TIM modulation on BsAb-driven T cell infiltration into tumors, their persistence, and in vivo anti-tumor response. Anti-GD2 BsAb and anti-HER2 BsAb built on IgG-[L]-scFv platform were tested against human cancer xenografts in BALB-*Rag2*^−/−^IL-2R-*γc*-KO (BRG) mice. Depleting antibodies specific for polymorphonuclear myeloid-derived suppressor cell (PMN-MDSC), monocytic MDSC (M-MDSC), and tumor associated macrophage (TAM) were used to study the role of each TIM component. Dexamethasone, an established anti-inflammatory agent, was tested for its effect on TIMs.

**Results:**

BsAb-driven T cells recruited myeloid cells into human tumor xenografts. Each TIM targeting therapy depleted cells of interest in blood and in tumors. Depletion of PMN-MDSCs, M-MDSCs, and particularly TAMs was associated with enhanced T cell infiltration into tumors, significantly improving tumor control and survival in multiple cancer xenograft models. Dexamethasone premedication depleted monocytes in circulation and TAMs in tumors, enhanced BsAb-driven T cell infiltration, and anti-tumor response with survival benefit.

**Conclusion:**

Reducing TIMs markedly enhanced anti-tumor effects of BsAb-based T cell immunotherapy by improving intratumoral T cell infiltration and persistence. TAM depletion was more effective than PMN- or M-MDSCs depletion at boosting the anti-tumor response of T cell engaging BsAb.

**Supplementary Information:**

The online version contains supplementary material available at 10.1186/s13045-021-01156-5.

## Introduction

T cell-based immunotherapy has emerged as one of the most promising therapeutic modalities for refractory or relapsed cancers. Yet, major hurdles still remain. Besides their inability to reach and penetrate solid tumors, T cells tend to be exhausted or inhibited by immunosuppressive tumor microenvironment (TME) [1]. TME plays a key role in tumor development, growth, and metastasis [2], and the reciprocal interactions between cancer cells and TME promote cancer cell stemness and induce metabolic derangement by creating hypoxic and acidic environment [3]. The immune-hostile TME increases cancer-associated neutrophils and recruits myeloid-derived suppressor cells (MDSCs) and M2-polarized tumor associated macrophages (TAMs) [4, 5]. These tumor-infiltrating myeloid cells (TIMs) constitute a heterogeneous population of cells marked by diversity, plasticity, and immaturity and function as potent mediators of immune suppression, tumor angiogenesis, and tumor cell metastases [6–8]. In murine tumor models, TIMs are commonly classified as either ‘pro-tumoral’ M2 TAMs or MDSCs. MDSCs are further subdivided into 2 major groups: CD11b^+^Ly6G^+^Ly6C^lo^ polymorphonuclear MDSC (PMN-MDSC) and CD11b^+^Ly6G^−^Ly6C^hi^ monocytic MDSC (M-MDSC) [9–14]. MDSCs inhibit T cell metabolism by hoarding key amino acids [15–17], modulate T cell homing by cleaving L-selectin [4, 18], and prevent T cell activation through PD-L1 upregulation especially when hypoxic [19]. Immunosuppressive M2 TAMs promote T cell anergy via increased nitric oxide (NO) and decreased arginine under hypoxic conditions [20]. Modulating these TIMs has the potential to improve anti-tumor response of T cell-based cancer immunotherapies.

We previously demonstrated that the IgG-[L]-scFv-formatted T cell engaging bispecific antibody (T-BsAb) was highly effective in driving human T cells into human tumor xenografts [21]. Here, we explore the effects of myeloid cell depleting strategies targeting TAM, PMN-MDSC, or M-MDSC, as well as dexamethasone, on T-BsAb mediated immunotherapy, with specific emphasis on T cell infiltration into tumors and in vivo anti-tumor activity.

## Methods

### Construction and expression of BsAbs

The construction of the GD2-BsAb was described previously [22]. For each BsAb, scFv of huOKT3 was fused to the C-terminus of the light chain of human IgG1 via a C-terminal (G_4_S)_3_ linker [23]. N297A and K322A on Fc were generated with site-directed mutagenesis via primer extension in polymerase chain reactions [24]. The nucleotide sequence encoding each BsAb was synthesized by GenScript and was subcloned into a mammalian expression vector. BsAb was produced using Expi293™ expression system (Thermo Fisher Scientific RRID:CVCL_D615) separately. Antibodies were purified with protein A affinity column chromatography. The purity of these antibodies was evaluated by size-exclusion high-performance liquid chromatography (SE-HPLC).

### Cell lines

Representative melanoma cell line M14 (UCLA-SO-M14, NCI-DTP Cat# M14, RRID:CVCL_1395), osteosarcoma cell line 143B (ATCC Cat# CRL-8303, RRID:CVCL_2270) and small cell lung cancer (SCLC) cell line NCI-N417 (ATCC Cat# CRL-5809, RRID:CVCL_1602) were used. All the cell lines used were authenticated by short tandem repeat profiling with the PowerPlex 1.2 System (Promega, Cat# DC8942), and periodically tested for mycoplasma infection using MycoAlert™ PLUS Mycoplasma Detection Kit (Lonza, Cat# LT07-318). The cells were cultured in RPMI1640 (Sigma) supplemented with 10% heat-inactivated fetal bovine serum (FBS, Life Technologies) at 37 °C in a 5% CO_2_ humidified incubator. The luciferase-labeled osteosarcoma cell line 143BLuc and melanoma cell line M14Luc were generated by retroviral infection with an SFG-GF Luc vector.

### BsAb treatment and depleting antibodies

10 µg of GD2-BsAb was given intravenously with 2 × 10^7^ of effector T cells or as ex vivo GD2-BsAb armed T cells (GD2-EATs). Ex vivo BsAb armed T cells (EATs) were generated as follows: T cells isolated from peripheral blood mononuclear cells (PBMCs) were activated with CD3/CD28 Dynabeads (GibcoTM, Cat#11132D) for 7 to 14 days in the presence of 30 IU/mL of IL-2. 2 × 10^7^ of activated T cells were armed with 10 µg of each BsAb for 20 min at room temperature [21]. After incubation, the T cells were washed with PBS twice. EATs were tested for cell surface density of BsAb using idiotype antibody and in vitro cytotoxicity against target antigens. Activated T cells or EATs were injected intravenously with 1000 IU of IL-2 given subcutaneously. For depletion of tumor infiltrating myeloid cells, 100 µg of anti-mouse GR1((Bio X Cell Cat# BE0320, RRID:AB_2819047), 100 µg of anti-mouse Ly6G (Bio X Cell Cat# BE0075-1, RRID:AB_1107721), 100 µg of anti-mouse Ly6C antibody (Bio X Cell Cat# BE0203, RRID:AB_2687696), 100 µg of anti-mouse CSF-1R (CD115) antibody (Bio X Cell Cat# BE0213, RRID:AB_2687699), or 10µL of clodronate liposome (Liposoma B.V., Cat#SKU-C005) was given iv one day before, and dexamethasone sodium phosphate were given ip one hour before, each BsAb or EAT injection.

### Anti-tumor effect in human tumor xenografts

All animal procedures were performed in compliance with Memorial Sloan Kettering Cancer Center’s institutional Animal Care and Use Committee (IACUC) guidelines. In vivo anti-tumor response was evaluated using cancer cell line- or patient-derived xenografts (CDXs or PDXs). Target antigen (+) patient-derived xenograft (PDX) was established from fresh surgical specimens with MSKCC IRB approval. Tumor cells suspended in Matrigel (Corning Corp, Cat#354234) or PDXs were implanted in the right flank of 6–10-week-old BALB-Rag2-/-IL-2R-γc-KO (BRG) mice (Taconic Biosciences). To avoid biological variables, only female mice were used for in vivo experiments. Treatment was initiated after tumors were established, average tumor volume of 100 mm^3^ when measured using TM900 scanner (Piera, Brussels, BE). Before treatment, mice with small tumors (< 50 mm^3^) or infection signs were excluded from the experiments, and the included mice were randomly assigned to each group. Tumor growth curves and overall survival was analyzed, and the overall survival was defined as the time from start of treatment to when tumor volume reached 2000 mm^3^. To define the well-being of mice, CBC analyses, changes in body weight, behavior and physical appearance were monitored. All animal experiments were repeated twice more with different donor’s T cells to ensure that our results were reliable.

### T cell transduction

T cells isolated from peripheral blood (PB) were stimulated with Dynabeads™ Human T-Activating CD3/CD28 beads (GibcoTM, Cat#11132D) for 24 h. T cells were transduced with retroviral constructs containing tdTomato and click beetle red luciferase in RetroNectin-coated 6-well plates in the presence of IL-2 (100 IU/ml) and protamine sulfate (4 μg/mL). Transduced T cells were cultured for 8 days before being used in animal experiments.

### Bioluminescence imaging

Mice were anesthetized and imaged after intravenous injection of 3 mg of D-luciferin (Gold Biotechnology, Cat# LUCK-100) on different days post T cell injection. Images were acquired using IVIS Spectrum CT In Vivo Imaging System (Caliper Life Sciences). Bioluminescence images were overlaid onto photographs, and regions of interest (ROI) were drawn based on the location and contour of tumor using Living image 2.60 (Xenogen) to quantify bioluminescence emission (BLI, photon flux/sec).

### Flow cytometry analysis and cytokine assay

For blood and tumor samples from mice, the following antibodies were purchased from BioLegend: anti-human CD45-APC (BioLegend Cat# 304011, RRID:AB_314399), anti-human CD45-Brilliant Violet 421™ (BioLegend Cat# 304032, RRID:AB_2561357), anti-human CD4-APC (BioLegend Cat# 300514, RRID:AB_314082), anti-human CD4-PE/Cyanine7 (BioLegend Cat# 300512, RRID:AB_314080), anti-human CD8-FITC (BioLegend Cat# 980908, RRID:AB_2888883), anti-human CD8-Brilliant Violet785™ (BioLegend Cat# 344739, RRID:AB_2566201), anti-mouse CD45-APC (BioLegend Cat# 103111, RRID:AB_312976), anti-mouse CD45-Brilliant Violet 711™ (BioLegend Cat# 103147, RRID:AB_2564383), anti-mouse/human CD11b-Brilliant Violet 570™ (BioLegend Cat# 101233, RRID:AB_10896949), anti-mouse/human CD11b-PE (BioLegend Cat# 101208, RRID:AB_312791), anti-mouse/human CD11b-PE/Cyanine7 (BioLegend Cat# 101215, RRID:AB_312798), anti-mouse Ly6G-FITC (BioLegend Cat# 127606, RRID:AB_1236494), anti-mouse Ly6G-PerCP/Cyanine5.5 (BioLegend Cat# 127616, RRID:AB_1877271), anti-mouse Ly-6C-PerCP/Cyanine5.5 (BioLegend Cat# 128012, RRID:AB_1659241), anti-mouse F4/80-PE/Cyanine7 (BioLegend Cat# 123114, RRID:AB_893478), anti-mouse F4/80-PE (BioLegend Cat# 123110, RRID:AB_893486).

### Immunohistochemistry and immunofluorescence staining

Immunohistochemistry (IHC) and immunofluorescence (IF) staining were performed at the MSK Molecular Cytology Core Facility using Discovery XT processor (Ventana Medical Systems) as previously described [22]. Tumor samples were fixed and embedded in paraffin. Anti-human CD45, anti-human CD3, anti-human CD4, anti-human CD8, anti-human FoxP3, anti-mouse CD45, anti-mouse CD11b, anti-mouse CD68, and anti-mouse IBA1 antibodies were used, which were followed by biotinylated secondary antibody. The detection was performed using a DAB detection kit (Ventana Medical Systems) or Alexa Fluor™ 488 or 568 Tyramide Reagent (Invitrogen). IHC images were captured from tumor sections using a Nikon ECLIPSE Ni-U microscope and NIS-Elements 4.0 imaging software. IF images were captured using Leica Inverted Confocal SP8 and processed with Imaris (Bitplane). Antigen positive cells were counted with Qupath 0.1.2.

### Positive pixel count analysis

IHC slides were scanned (Aperio ScanScope XT) and analyzed by comparing positive pixel counts (Aperio Technologies). For analyzing tumor infiltrating lymphocytes, the largest area of intact tumor tissue was included, and oblique sections were avoided. Each slide was visually inspected to ensure specificity and sensitivity of antibody staining. After positive pixel count analysis was run, analyzed slides were examined to confirm that positively identified pixels were consistent with lymphocyte staining and not background staining. Percentages were calculated as the total number of positive pixels divided by the total number of pixels (% positive pixels/total pixels).

### Statistical analysis

In vivo anti-tumor effect was compared by area under curve (AUC) and survival curve analyses. TILs were quantified using AUC of BLIs. Differences between samples indicated in the figures were tested for statistical significance by two-tailed Student’s t-test for two sets of data while one-way ANOVA with Tukey’s post hoc test was used to among three or more sets of data. All statistical analyses were performed using GraphPad Prism V.8.0 for Windows (GraphPad Software, La Jolla, CA, www. graphpad. com). *P* value < 0.05 was considered statistically significant. Asterisks indicate that the experimental *P* value is statistically significantly different from the associated controls at * *P* < 0.05; ** *P* < 0.01; *** *P* < 0.001, **** *P* < 0.0001.

### Results

### T-BsAb transformed immunologically ‘cold tumors’ to ‘hot tumors’

**Fig. 1:**
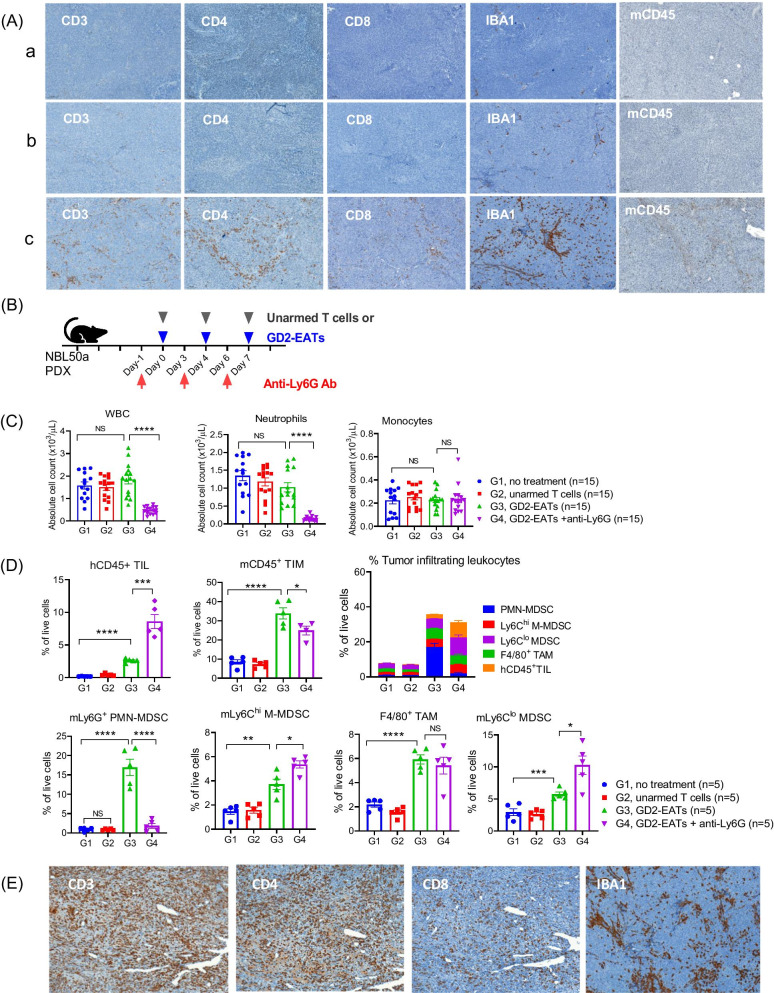
The effects of granulocyte depletion on bispecific antibody (BsAb) directed T cell immunotherapy. **A** Neuroblastoma patient-derived xenografts (PDXs) treated with GD2-BsAb armed T cells (GD2-EATs) or unarmed T cells were compared with untreated tumors. All tumors were harvested on day 10 after the start of treatment. Immunohistochemical (IHC) staining of the tumor sections by anti-human CD3 antibody (× 10), anti-human CD4 antibody (× 10), anti-CD8 antibody (× 10), anti-mouse IBA1 antibody (× 10), and anti-mouse CD45 antibody (x 10). a, no treatment; b, unarmed T cells; c, GD2-EATs. **B** Neuroblastoma PDX bearing mice were treated by GD2-EATs with anti-Ly6G antibody to deplete granulocytes. **C** The complete blood count (CBC) test was done and compared among groups. **D** Tumors harvested on day 10 were analyzed by flow cytometry (Additional file 1: Fig. S3), and the frequencies of each tumor infiltrating leukocytes were compared among groups. **E** The PDXs treated with GD2-EATs plus anti-Ly6G antibody were harvested on day 10 after the start of treatment and were stained by anti-human CD3 antibody (× 10), anti-human CD4 antibody (× 10), anti-CD8 antibody (× 10), anti-mouse IBA1 antibody (× 10)

We previously demonstrated that ex vivo armed T cells with IgG-[L]-scFv platformed BsAb (EATs) infiltrated solid tumors rapidly and persisted for weeks, leading to complete tumor ablation [25]. Here we administered unarmed T cells or T cells armed with GD2-BsAb (GD2-EATs) into mice xenografted with neuroblastoma PDX (Additional file 1: Fig. S1A). Tumors were analyzed by flow cytometry on day 10 post-treatment (Additional file 1: Fig. S1B). While unarmed T cell-treated or untreated tumors had few leukocytes typical for ‘cold tumors’, those treated with GD2-EATs had plenty of leukocytes, including human T cells and mouse-derived myeloid cells—all characteristics of ‘hot tumors’. In contrast to tumors without treatment or treated with unarmed T cells showing few TIMs, tumors treated with GD2-EATs were infiltrated with higher frequencies of mCD45^+^TIMs, comprising 50% of PMN-MDSCs (CD11b^+^Ly6G^+^Ly6C^lo^), 20% of TAMs (CD11b^+^Ly6G^−^Ly6C^lo^F4/80^+^), 11% of M-MDSCs (CD11b^+^Ly6G^−^Ly6C^hi^), and 17% of Ly6C^lo^ MDSCs (CD11b^+^Ly6G^−^Ly6C^lo^F4/80^−^), respectively. When tumors were studied by IHC (Fig. 1A), the tumors in control groups (unarmed T cells or no treatment) showed few leukocytes, whereas GD2-EAT-treated tumors had diffuse T cell infiltration along with strong positivity for mouse myeloid cell markers, i.e., mCD45 and IBA1 (ionized calcium-binding adaptor molecule-1) staining. These findings were confirmed in 143B osteosarcoma CDX model (Additional file 1: Fig. S2). The tumors treated with GD2-BsAb plus human PBMCs presented robust CD11b^+^ TIM infiltration with diffuse CD3+ T cell infiltration, contrasting with the tumors treated with PBMCs alone which had few leukocyte infiltrates.

### TIM depletion enhanced anti-tumor effect of T-BsAb

#### Granulocyte depletion

Although T-BsAb successfully transformed ‘cold tumors’ to immunologically ‘hot tumors’, not all tumors responded to treatment. To test if removing TIMs could enhance anti-tumor response of T-BsAb, we selectively depleted each TIM and studied the effect on BsAb-driven T cell trafficking, infiltration, and in vivo anti-tumor response.

In order to deplete PMN-MDSCs, anti-Ly6G antibody was administered one day before each GD2-EAT treatment (Fig. 1B). After 2 doses of anti-Ly6G antibody and GD2-EATs, peripheral blood (PB) was collected for CBC (Fig. 1C). Anti-Ly6G antibody successfully removed neutrophils but did not affect monocytes in the circulation. The neuroblastoma PDXs were harvested on day 10 post-treatment and analyzed by flow cytometry (Fig. 1D and Additional file 1: Fig. S3). When compared to the tumors treated with GD2-EATs alone, anti-Ly6G combination significantly reduced the frequency of mLy6G^+^PMN-MDSCs (*P* < 0.0001) without affecting mCD1b^+^F4/80^+^ TAMs. This was accompanied by a reciprocal increase in human T cells, mLy6C^hi^ M-MDSCs, and mLy6C^lo^ MDSCs (*P* = 0.039, *P* = 0.014, and *P* < 0.0001, respectively). IHC staining of tumors confirmed the inverse relationship of granulocyte depletion and enhanced T cell infiltration, for both CD4^+^ and CD8^+^ T cells (Fig. 1E). Macrophage-specific protein IBA1 staining of tumors did not show significant difference compared to tumors treated with GD2-EATs alone. To quantify T cell infiltration in tumors and characterize T cell populations, positive pixel count analysis was performed on IHC slides stained with antibodies against CD3, CD4, and CD8 (Supplementary Table S1) [26]. Tumors treated with GD2-EATs plus anti-Ly6G showed 3.8-fold increase of CD3, 5.8-fold increase of CD4, and 4.8-fold increase of CD8 T cell infiltration compared to GD2-EATs alone (*P* = 0.005, *P* = 0.02, and *P* = 0.003, respectively).

The effect of granulocyte depleting antibodies was also tested in 143B osteosarcoma cell line xenograft mouse model (Additional file 1: Fig. S4A). Anti-GR1 or anti-Ly6G antibody was administered with GD2-EATs or unarmed T cells. CBC analyses on day 5 showed that anti-Ly6G or anti-GR1 antibody significantly decreased neutrophil count in the circulation, while anti-GR1 depleted monocytes as well (Additional file 1: Fig. S4B). Tumors were harvested and analyzed on day 60 post-treatment (Additional file 1: Fig. S4C). Anti-GR1 or anti-Ly6G antibody significantly increased the frequencies of hCD45^+^ or hCD8^+^ TILs by flow cytometry, confirming the effect by anti-human CD3 IHC staining of tumors (Additional file 1: Fig. S4D).

**Fig. 2:**
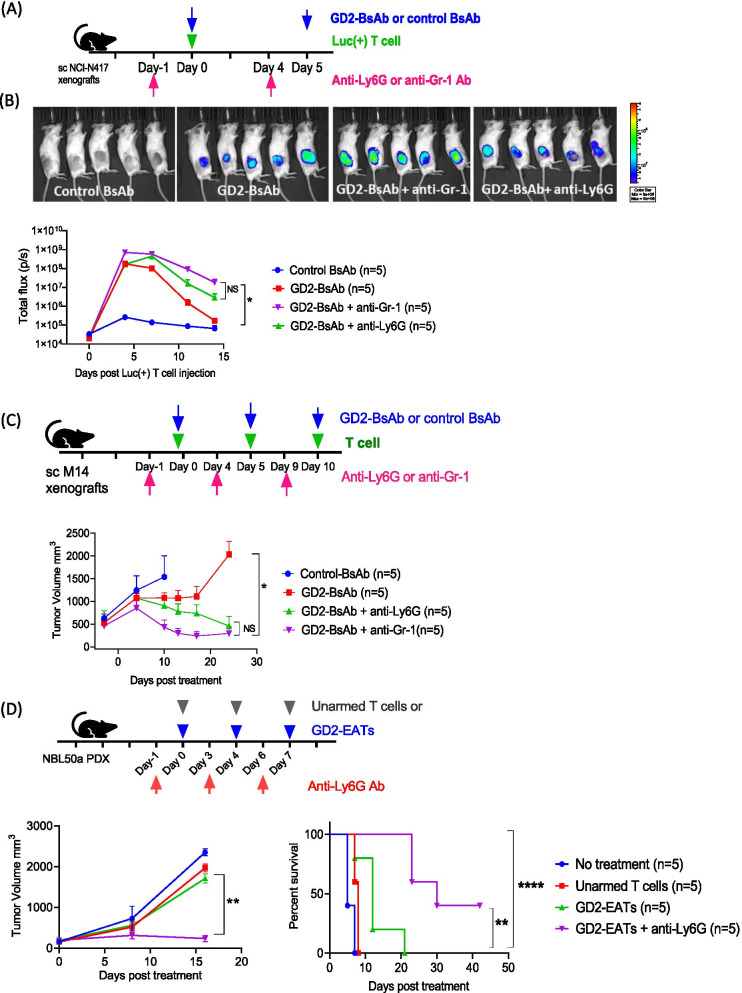
The effects of granulocyte depletion on BsAb directed T cell trafficking and in vivo anti-tumor response. **A** Luciferase transduced T cells [Luc(+) T cells] were administered with GD2-BsAb and anti-GR1 or anti-Ly6G antibody to treat small cell lung cancer cell line (NCI-N417) xenografts. Luc(+) T cells were administered intravenously (iv) on day 0, and GD2-BsAbs were iv administered on day 0 and day 5 separately. Anti-Ly6G or anti-GR1 antibodies were administered on day -1 and day 4 intraperitoneally. **B** Bioluminescence of Luc(+) T cells infiltrated into tumors were monitored. The bioluminescence images on day 4 and quantification of the bioluminescence in the lesions of tumor. Control BsAb, Luc(+) T cells plus control BsAb; GD2-BsAb, Luc(+) T cells plus GD2-BsAb; GD2-BsAb plus anti-GR1, Luc(+) T cells plus GD2-BsAb plus anti-GR1 antibody; GD2-BsAb plus anti-Ly6G, Luc(+) T cells plus GD2-BsAb plus anti-Ly6G antibody. **C** In vivo anti-tumor response by GD2-BsAb and granulocyte depleting antibodies was tested against melanoma cell line (M14) xenografts. Control BsAb, Luc(+) T cells plus control BsAb; GD2-BsAb, Luc(+) T cells plus GD2-BsAb; GD2-BsAb+ anti-GR1, Luc(+) T cells plus GD2-BsAb plus anti-GR1 antibody; GD2-BsAb+ anti-Ly6G, Luc(+) T cells plus GD2-BsAb plus anti-Ly6G antibody. **D** In vivo anti-tumor effect of GD2-EATs with anti-Ly6G antibody was tested against neuroblastoma patient-derived xenografts (PDXs), and long-term survival was analyzed

To study the effect of granulocyte depletion on BsAb-driven T cell trafficking, luciferase labeled T cells [Luc(+) T cells] and soluble GD2-BsAb were administered to mice xenografted with GD2(+) small cell lung cancer (SCLC) cell line NCI-N417 one day after iv treatment of anti-Ly6G or anti-GR1 antibody (Fig. 2A). Luciferase signals in the tumors were monitored, and mean bioluminescence intensity (total flux, photon/sec) of TILs was compared among groups (Fig. 2B). In contrast to control BsAb, GD2-BsAb successfully drove Luc(+) T cells into xenografts, and the addition of anti-GR1 or anti-Ly6G antibody amplified the bioluminescent intensity (BLI) in tumors more than two-fold above those treated with GD2-BsAb. In addition, these granulocyte depleting antibodies delayed the loss of bioluminescence from the tumors. BLI by day 11 has dropped to 1% of its peak in the GD2-BsAb group. In contrast, with the addition of anti-Ly6G or anti-GR1 antibody, the BLI by day 11 was 10 to 50-fold higher compared to GD2-BsAb (*P* = 0.009). The enhanced BLI of TILs by depleting granulocytes was confirmed again in neuroblastoma PDX model (Additional file 1: Fig. S5). Area under curves (AUC) of the BLI analyzed to estimate the total quantity of TILs increased by an average of 2.9-fold to 5.5-fold in the anti-Ly6G or anti-GR1 combination treatment groups, respectively (Supplementary Table S2).

The enhancement of T cell infiltration and persistence in tumors by granulocyte depletion translated to a substantial improvement in anti-tumor effect in vivo. In contrast to control-BsAb where tumors grew unabated, GD2-BsAb treatment suppressed tumor growth, and the anti-tumor response against melanoma cell line (M14) xenografts was much enhanced by granulocytes depletion (*P* = 0.01) (Fig. 2C). The in vivo anti-tumor effect was also tested in neuroblastoma PDX (Fig. 2D) and osteosarcoma CDX models (Additional file 1: Fig. S4E). Anti-Ly6G antibody improved in vivo efficacy of GD2-EATs against neuroblastoma PDXs (*P* = 0.003) and prolonged survival (*P* = 0.002). Osteosarcoma xenografts also demonstrated the benefit of anti-GR1 or anti-Ly6G antibody, with improved tumor control and overall survival compared to GD2-EATs alone (*P* = 0.024).

#### Monocyte depletion

**Fig. 3:**
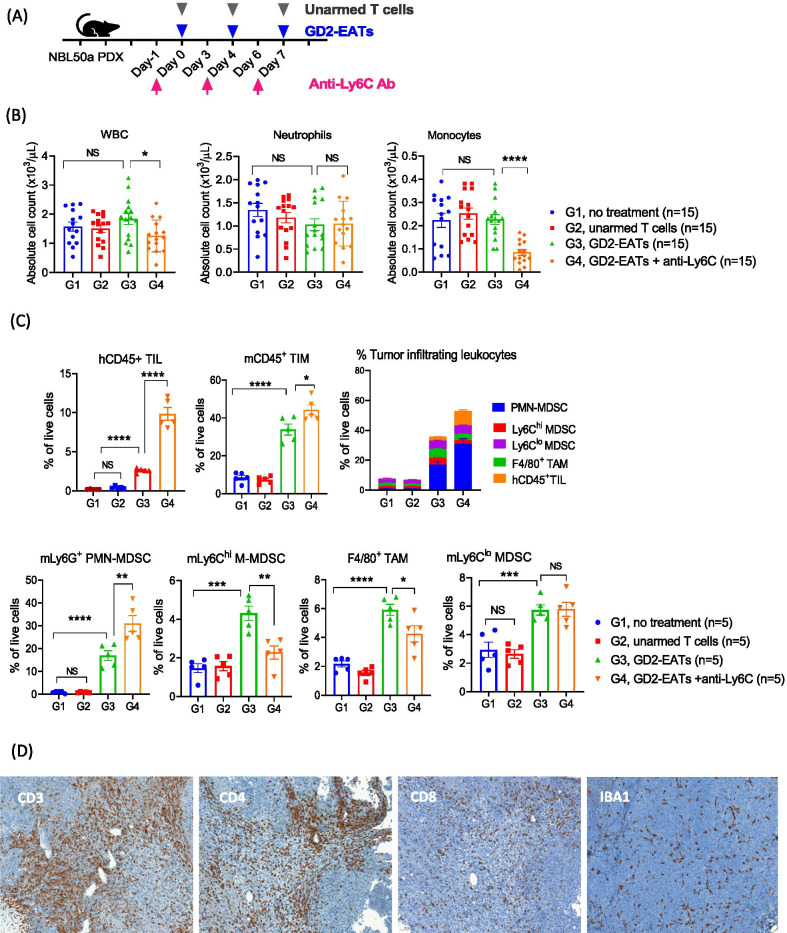
The effects of monocyte depletion on BsAb directed T cell immunotherapy. **A** Neuroblastoma PDX bearing mice were treated by GD2-BsAb armed T cells (GD2-EATs) with anti-Ly6C antibody to deplete monocytes. **B** Peripheral blood (PB) was analyzed by CBC and compared among groups. **C** Tumors harvested on day 10 were analyzed by flow cytometry (Additional file 1: Fig. S3), and the frequencies of each tumor infiltrating leukocytes were compared among groups. **D** Immunohistochemical (IHC) staining of tumor sections by anti-human CD3 antibody (× 10), anti-human CD4 antibody (× 10), anti-CD8 antibody (× 10), and anti-mouse IBA1 antibody (× 10). The tumors were harvested on day 10 after the start of treatment

Anti-Ly6C antibody was applied to deplete M-MDSC one day before GD2-EATs injection in a neuroblastoma PDX mouse model (Fig. 3A). After 2 doses of anti-Ly6C antibody and GD2-EATs, PB was collected for CBC analyses (Fig. 3B). Anti-Ly6C antibody successfully depleted monocytes without affecting neutrophils in the circulation. Tumors were harvested on day 10 post-treatment and analyzed by flow cytometry (Fig. 3C and Additional file 1: Fig. S3). After anti-Ly6C antibody treatment, the frequencies of mLy6C^hi^ M-MDSCs and F4/80^+^ TAMs decreased when compared to GD2-EATs alone (*P* = 0.004 and *P* = 0.04, respectively). While the frequency of mLy6G^+^ PMN-MDSC increased, that of Ly6C^lo^ MDSC did not change. In the presence of anti-Ly6C antibody, GD2-EAT treated tumors showed more robust hCD45^+^ TIL infiltration than tumors treated with GD2-EATs alone. IHC staining using anti-human CD3, CD4, and CD8 antibody confirmed the increase of TILs  when treated with GD2-EATs in the presence of anti-Ly6C antibody (Fig. 3D). Tumors treated with GD2-EATs plus anti-Ly6C showed 2.3-fold increase of CD3, 3.2-fold increase of CD4, and fourfold increase of CD8 T cell infiltration compared to GD2-EATs alone by positive pixel count analysis (*P* = 0.023, *P* = 0.083, and *P* = 0.1522, respectively) (Supplementary table S1).

These effects of anti-Ly6C antibody were reproduced in the osteosarcoma tumor model (Additional file 1: Fig. S6A). CBC analyses on day 5 showed a significant decrease of monocyte count (Additional file 1: Fig.S6B). Tumors were harvested on day 60 post-treatment and analyzed for T cell infiltration. The frequency of hCD45^+^TIL or hCD8^+^ TIL was significantly higher after treatment of Ly6C and GD2-EATs combination compared to GD2-EATs alone by flow cytometry (*P* = 0.044 and *P* = 0.045, respectively) (Additional file 1: Fig. S6C). CD3 IHC staining also showed increased intratumoral GD2-EAT infiltration following anti-Ly6C antibody (Additional file 1: Fig. S6D).

**Fig. 4:**
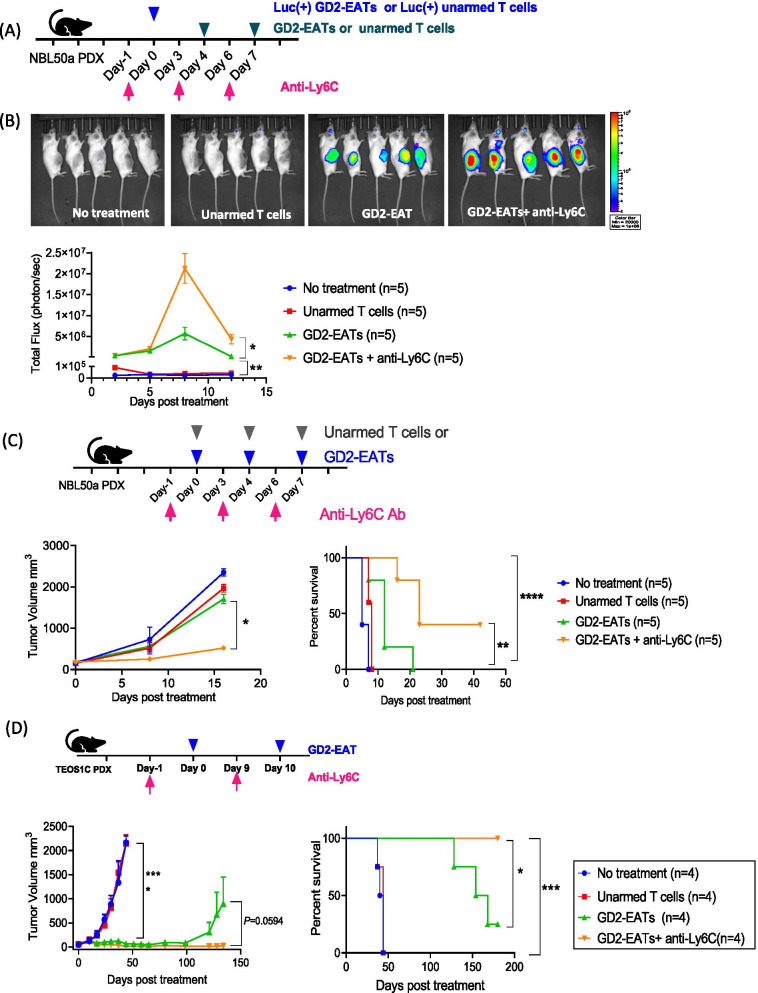
The effects of monocyte depletion on BsAb directed T cell trafficking and in vivo anti-tumor response. **A** Luciferase transduced T cells [Luc(+) T cells] or Luciferase transduced GD2-BsAb armed T cells [Luc(+) GD2-EATs] were administered with anti-Ly6C antibody to the mice bearing neuroblastoma patient-derived xenograft (PDX). **B** Bioluminescence in the lesions of tumor was monitored. The bioluminescence images on day 7 and quantification of the bioluminescence in the lesions of tumor. **C** In vivo anti-tumor response by GD2-EATs with anti-Ly6C antibody was tested against neuroblastoma PDXs. **D** In vivo anti-tumor effect of GD2-EATs with anti-Ly6C antibody was tested against osteosarcoma PDXs, and long-term survival was analyzed

To study the effect of M-MDSCs depletion on T cell trafficking, anti-Ly6C antibody was administered with Luc(+) GD2-EATs in neuroblastoma PDX model (Fig. 4A). Unlike the Luc(+) unarmed T cells which disappeared following a transit sequestration in the lungs, the Luc(+) GD2-EATs efficiently trafficked to the tumor sites and persisted longer, as evidenced by their bioluminescence (Fig. 4B). Anti-Ly6C antibody significantly enhanced peak BLI in the tumors (*P* = 0.02) while delaying their disappearance, with bioluminescence remained 20-fold higher when compared to GD2-EATs alone on day 12 (*P* = 0.006). Total quantity of TILs assessed by AUC of the BLI increased by 3.5-fold compared to GD2-EATs alone (Supplementary Table S2).

Depletion of M-MDSCs was associated with significantly improved tumor control as well. Neuroblastoma PDXs treated by GD2-EATs combined with anti-Ly6C antibody showed improved tumor control compared to GD2-EATs alone (*P* = 0.04) and enhanced survival (*P* = 0.008) (Fig. 4C). In a separate set of experiments using osteosarcoma PDXs, while 3 of 4 mice treated with GD2-EATs experiencing tumor relapse, all 4 mice treated with GD2-EATs plus anti-Ly6C antibody showed a long-term remission (> 180 days post treatment), demonstrating the benefit of depleting M-MDSCs (*P* = 0.04), (Fig. 4D). The benefit of anti-Ly6C antibody was verified again in osteosarcoma cell line derived xenografts (CDXs) (Additional file 1: Fig. S6), where anti-Ly6C antibody increased intratumoral infiltration of GD2-EATs when compared with GD2-EATs alone, thereby translating to enhanced in vivo anti-tumor response (*P* = 0.006).

#### Macrophage depletion

**Fig. 5:**
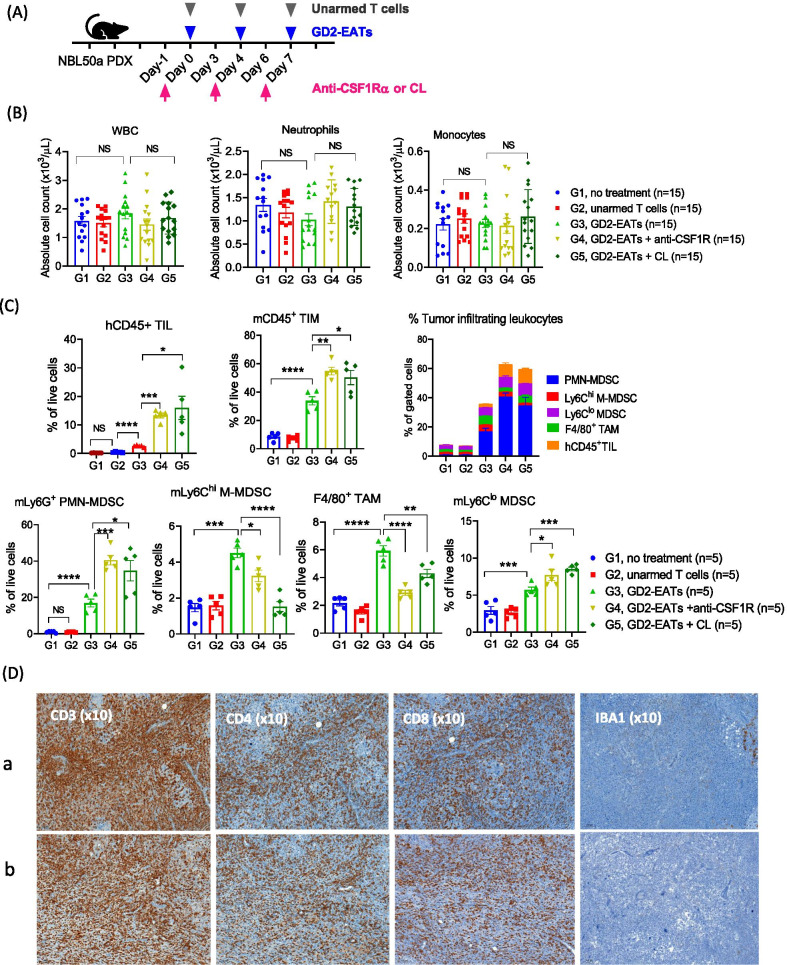
The effects of macrophage depletion on BsAb directed T cell immunotherapy. **A** Neuroblastoma PDX bearing mice were treated by GD2-BsAb armed T cells (GD2-EATs) with anti-CSF1R antibody or clodronate liposome (CL) to deplete macrophages. **B** Peripheral blood (PB) was analyzed by CBC and compared among groups. **C** Tumors harvested on day 10 were analyzed by flow cytometry (Additional file 1: Fig. S3), and the frequencies of each tumor infiltrating leukocytes were compared among groups. **D** Immunohistochemical (IHC) staining of tumor sections by anti-human CD3 antibody (× 10), anti-human CD4 antibody (× 10), anti-CD8 antibody (× 10), and anti-mouse IBA1 antibody (× 10). The tumors were harvested on day 10 after the start of treatment

We treated mice with either clodronate liposome (CL) or anti-CSF1R antibody to deplete macrophages and investigated their effect on T-BsAb treatment. After 2 doses of each treatment, macrophages were depleted from the liver or the spleen (Additional file 1: Fig. S7A and Fig. S7B). Neuroblastoma PDXs were treated with GD2-EATs and anti-CSF1R antibody or CL (Fig. 5A). CBC analyses after 2 doses of anti-CSF1R antibody or CL did not show significant differences among treatment groups: anti-CSF1R antibody or CL did not affect neutrophils or monocytes in the circulation (Fig. 5B). When tumors were analyzed on day 10 (Fig. 5C and Additional file 1: Fig. S3), anti-CSF1R antibody and CL significantly increased hCD45^+^ TILs and mCD45^+^ TIMs by flow cytometry. While the tumors showed a marked reduction of CD11b^+^F4/80^+^ TAMs and Ly6C^hi^ M-MDSCs, there was an increase in Ly6G^+^ PMN-MDSCs and Ly6C^lo^ MDSCs. IHC staining of tumors on day 10 showed that macrophage depletion greatly promoted BsAb-driven T cell infiltration reversely to the decrease of IBA-1(+) macrophages (Fig. 5D). The tumors treated with GD2-EATs plus anti-CSF1R showed 4.7-fold increase of CD3, 5.3-fold increase of CD4, and 17.8-fold increase of CD8 T cell infiltration compared to GD2-EATs alone (*P* = 0.009, *P* = 0.028, and *P* = 0.026, respectively); the tumors treated with GD2-EATs plus CL presented a 4.4-fold increase of CD3, 5.8-fold increase of CD4, and 15-fold increase of CD8 T cell infiltration by positive pixel count analyses (*P* < 0.0001, *P* = 0.009, and *P* = 0.0074, respectively), (Supplementary Table S1). In addition, the tumors treated with GD2-EATs plus anti-CSF1R antibody or CL showed a significant  increase in CD8 to CD4 TIL ratios compared to GD2-EATs alone (*P* = 0.001 and *P* = 0.0001, respectively). The enhancement of T cell infiltration by anti-CSF1R antibody or CL was also observed in 143B osteosarcoma CDXs (Additional file 1: Fig. S7C) and M14 melanoma CDXs (Additional file 1: Fig. S7D).

**Fig. 6:**
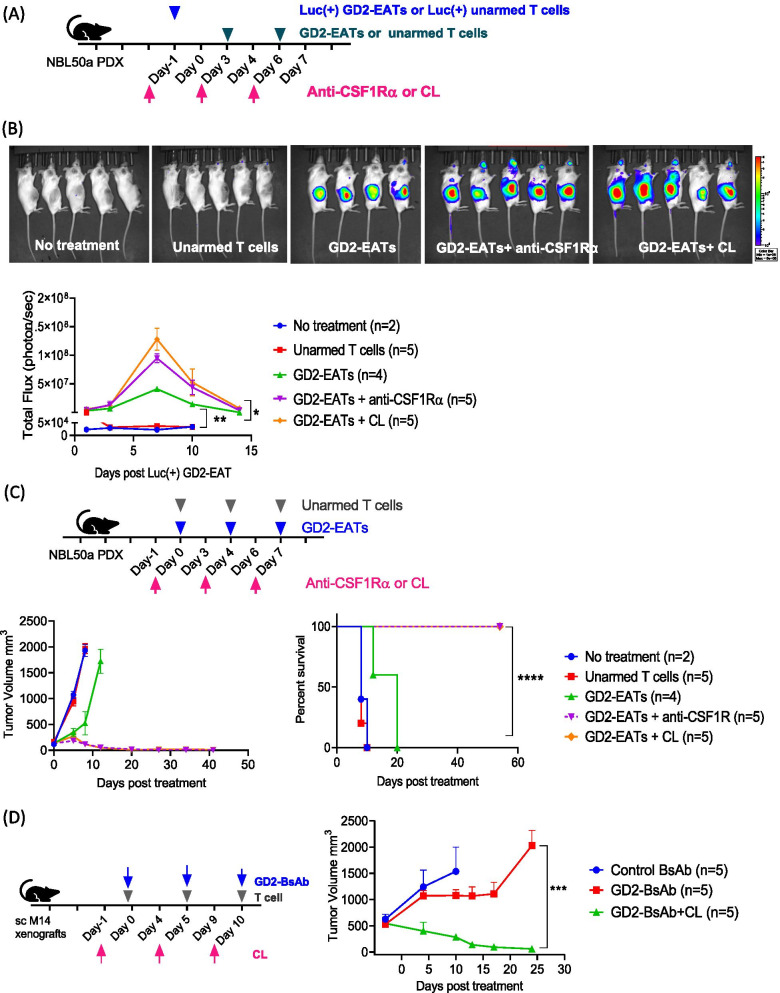
The effects of macrophage depletion on BsAb directed T cell trafficking and in vivo anti-tumor response. **A** Luciferase transduced T cells [Luc(+) T cells] or Luciferase transduced GD2-BsAb armed T cells [Luc(+) GD2-EATs] were administered with anti-CSF1R antibody or clodronate liposome (CL) to the mice bearing neuroblastoma patient-derived xenograft (PDX). **B** Bioluminescence in the lesions of tumor was monitored. The bioluminescence images on day 7 and quantification of the bioluminescence in the lesions of tumor. **C** In vivo anti-tumor response by GD2-EATs with anti-CSF1R antibody or CL was tested against neuroblastoma PDXs. **D** GD2-BsAb and T cells were separately administered intravenously with or without intraperitoneal CL. In vivo anti-tumor effect of GD2-BsAb with CL was tested against M14 melanoma cell line xenografts and compared with control BsAb or GD2-BsAb without CL

We next studied the effect of macrophage depletion on BsAb-driven T cell trafficking into solid tumors. Luc(+) GD2-EATs was administered with anti-CSF1R antibody or CL to neuroblastoma PDX bearing mice (Fig. 6A), and the BLI in tumors were quantified (Fig. 6B). Anti-CSF1R antibody or CL significantly increased the BLI in tumors treated with GD2-EATs (3.2-fold and 6.6-fold increase, respectively, Supplementary Table S2). The effect of CL on BsAb-driven T cell trafficking was also tested in SCLC CDXs (Additional file 1: Fig. S8A). With CL, the bioluminescence of T cell persisted through day 11, being 100-fold higher than those for GD2-BsAb alone (*P* = 0.02). The effect of anti-CSF1R antibody was also verified in an osteosarcoma PDX model (Additional file 1: Fig. S8B), where the mean BLI in tumors treated with HER2-EATs plus anti-CSF1R antibody was 40-fold higher than those for HER2-EATs alone on day 11 (*P* = 0.022). Based on the AUC of the BLI, the total quantity of TILs in tumors increased 4.5-fold and 3.6-fold in the presence of CL and anti-CSF1R antibody, respectively (Supplementary Table S2).

The enhanced T cell infiltration by macrophage depletion translated to improved anti-tumor response. Anti-CSF1R antibody or CL significantly enhanced tumor suppressive effect of GD2-EATs and prolonged survival without significant toxicity, surpassing the efficacy of G-MDSCs or M-MDSC depleting treatment (Fig. 6C). M14 xenografts treated with soluble GD2-BsAb plus CL showed complete regression despite large tumor volume at the beginning of treatment, contrasting to GD2-BsAb alone (without CL) where tumor grew unabated (Fig. 6D). The enhanced anti-tumor effect of macrophage depletion was confirmed in osteosarcoma PDX and 143B CDX models (Additional file 1: Fig. S9). CL or anti-CSF1R antibody significantly improved anti-tumor response of GD2-EATs without increasing toxicity, prolonging survival.

### Corticosteroid premedication to modulate TIMs

**Fig. 7:**
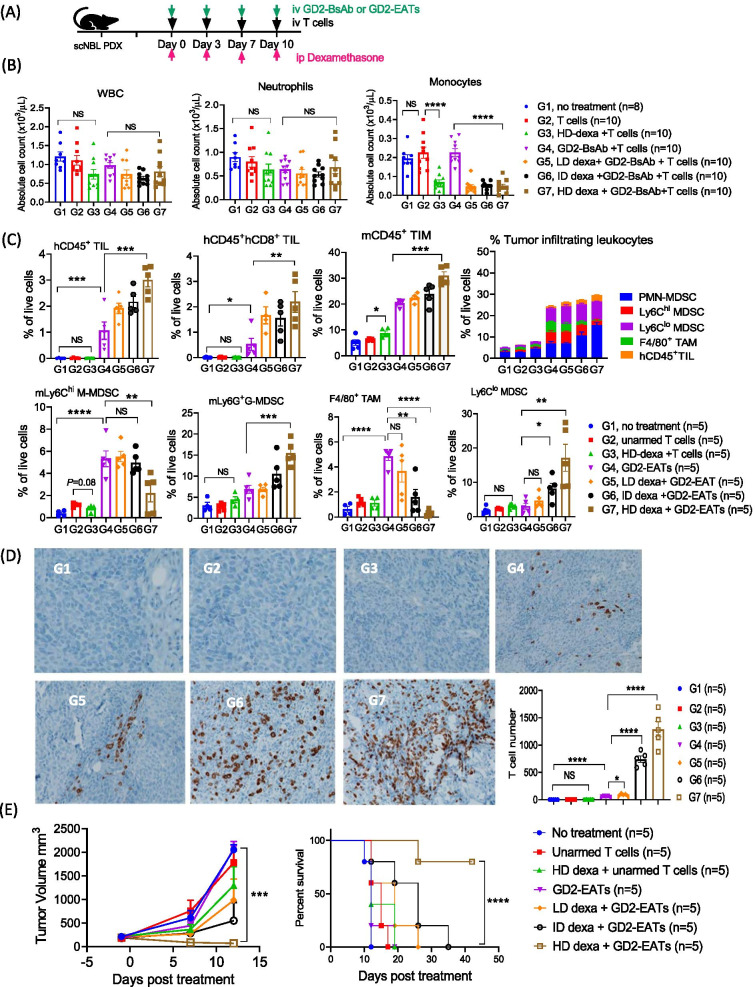
The effects of corticosteroids on BsAb directed T cell immunotherapy. **A** Neuroblastoma PDX bearing mice were treated by GD2-BsAb armed T cells (GD2-EATs) with increasing doses of dexamethasone. Dexamethasone was given 1 h before each GD2-EATs or unarmed T cells injection **B** Peripheral blood (PB) was analyzed by CBC and compared among groups. **C** Tumors harvested on day 25 were analyzed by flow cytometry (Additional file 1: Fig. S11), and the frequencies of each tumor infiltrating leukocytes were compared among groups. **D** Immunohistochemical (IHC) staining of tumor sections by anti-human CD3 antibody (× 20) on day 25 after the start of treatment. CD3^+^ T cell numbers were compared by Q-path analysis. G1, no treatment; G2, unarmed T cells; G3, unarmed T cells plus high-dose (HD) dexamethasone; G4, GD2-EATs; G5, GD2-EATs plus low-dose (LD) dexamethasone; G6, GD2-EATs plus intermediate-dose (ID) dexamethasone; G7, GD2-EATs plus high-dose (HD) dexamethasone. **E** In vivo anti-tumor response by GD2-EATs with increasing doses of dexamethasone was tested against neuroblastoma PDXs, and long-term survival was compared among groups

Corticosteroid was tested for their anti-inflammatory effect on TIM and TIL, since it is often used as a premedication to mitigate cytokine release during BsAb treatment. We tested increasing dose levels of dexamethasone [low-dose (LD); 2 mg/kg/dose, intermediate-dose (ID); 8 mg/kg/dose, and high-dose (HD); 32 mg/kg/dose], equivalent to 0.16 mg/kg to 2.6 mg/kg of human dose [27], as a premedication before each GD2-EAT treatment (Fig. 7A). Cytokine levels were analyzed at different time points post-treatment (Additional file 1: Fig. S10). Dexamethasone premedication decreased IL-2, IL-6, and TNF-α release from GD2-EATs, while IFN-*γ* levels were not significantly affected. After 2nd treatment dose, PB leukocytes were analyzed by CBC (Fig. 7B). Dexamethasone profoundly reduced monocytes but did not significantly affect neutrophils in the circulation. Dexamethasone also affected the frequencies of tumor infiltrating leukocytes by flow cytometry analyses (Fig. 7C and Additional file 1: Fig.S11). The increase in mCD45^+^ TIM and mLy6G^+^ PMN-MDSC was dexamethasone dose-dependent (*P* = 0.0001 and *P* = 0.0003, respectively). Ly6C^hi^ M-MDSCs were depleted by HD dexamethasone (*P* = 0.006), accompanied by an increase in the proportion of Ly6C^lo^ MDSC. Most notable was the decrease in the frequencies of mCD11b^+^F4/80^+^ TAMs following dexamethasone treatment (*P* = 0.0001). The depletion of TAMs was dose-dependent, being most apparent with HD dexamethasone (no dexamethasone vs. LD, *P* = 0.35; vs. ID, *P* = 0.002; vs. HD, *P* < 0.0001). Dexamethasone increased the frequency of hCD45+ TIL (*P* = 0.0007), and in particular CD8+ TIL in a dose-dependent fashion (*P* = 0.015), demonstrating an inverse correlation with TAMs. This effect of dexamethasone on intratumoral T cell infiltration was confirmed by IHC staining using anti-human CD3 antibody (Fig. 7D). Tumors treated with GD2-EAT plus ID or HD dexamethasone showed more abundant TILs compared to GD2-EATs alone (*P* < 0.0001).

The effect of dexamethasone premedication on BsAb-driven T cell trafficking was studied using an osteosarcoma PDX model (Additional file 1: Fig. S12). Intratumoral bioluminescence of Luc(+) GD2-EAT or Luc(+) unarmed T cell was quantified and compared among groups treated with increasing doses of dexamethasone. The BLI of tumors treated with GD2-EATs plus ID dexamethasone was tenfold higher than those for GD2-EATs alone on day 5, and the bioluminescence persisted throughout day 14. When estimated by BLI AUC, the total quantity of TILs increased 7.7-fold and 14-fold by LD- and ID-dexamethasone, respectively (Supplementary Table S2).

In vivo anti-tumor response also correlated with the BLIs in tumors and the frequencies of TIL. Dexamethasone did not compromise anti-tumor effect of GD2-BsAb against neuroblastoma PDXs (Fig. 7E and Additional file 1: Fig. S13A). Irrespective if administered as direct BsAb injection or as EATs, ID and HD dexamethasone enhanced the anti-tumor effect of GD2-BsAb, with significant improvement in survival (*P* < 0.0001). This favorable effect of dexamethasone on GD2-EATs was confirmed in another osteosarcoma PDX model. When LD (2 mg/kg) or ID (8 mg/kg) dexamethasone was premedicated, tumor control and survival improved in a dose-dependent manner with statistical significance (Additional file 1: Fig. S13B).

## Discussion

T-BsAbs built on the IgG-[L]-scFv platform, whether administered as stand-alone or as EATs are effective in driving T cells into solid tumors and inducing cytotoxic anti-tumor response against a number of cancer targets [22, 28–30]. Together with myeloid cells, these BsAb-driven T cells transformed immunologically ‘cold tumors’ into ‘hot tumors’. Using antibodies specific to deplete TIM subsets, we analyzed the PMN-MDSC, M-MDSC, and TAM following T-BsAb treatment. Based on selective myeloid cell depleting antibodies, we tested their role in human T cell infiltration into human tumors and in vivo anti-tumor response of BsAb treatment. Each of the myeloid cell inhibitors, including anti-Ly6G, anti-GR1, anti-Ly6C, anti-CSF1R antibodies, and CL decreased their respective mouse TIM subsets, enhancing T-BsAb-driven T cell infiltration and persistence, resulting in improved anti-tumor response. Dexamethasone premedication reduced circulating monocytes and TAMs in tumors in a dose-dependent manner, followed by a substantial improvement in intratumoral T cell infiltration and anti-tumor efficacy of BsAb treatment.

When we compared anti-tumor benefit from depletion of each myeloid cell, the most effective was macrophage depleting strategies. Anti-CSF1R antibody or CL significantly reduced the frequencies of not only mCD11b^+^F4/80^+^ TAM but also mLy6C^hi^ M-MDSC compared to the treatment of GD2-EATs alone, while markedly increasing the frequencies of intratumoral hCD45^+^ T cells. IHC staining of neuroblastoma PDXs showed that macrophage depletion significantly increased not only CD3 T cell infiltration but also CD8 to CD4 TIL ratios compared to GD2-EATs, while anti-Ly6G or anti-Ly6C antibody did not affect this ratio. TAM is one of the formidable obstacles to T cell immunotherapy, especially for solid tumors. While M1 macrophages play a role in human immunity by producing proinflammatory cytokines with strong phagocytic function, TAMs, recruited in TME, not only lack the function of phagocytizing tumor cells but also help tumor cells escape from immune surveillance and metastasize by stimulating angiogenesis and protecting tumor cells from cytotoxic T cells or NK cells [31–33]. In addition, TAMs are reprogrammed towards an iron-releasing phenotype, characterized by higher expression of the iron exporter ferroportin and iron transporter lipocalin-2, which promote tumor progression [34]. CD8^+^ cytotoxic T cells induce ferroptosis in tumor cells [35], and T cell-based immunotherapies including chimeric antigen receptor (CAR) or T-BsAb are likely to act through ferroptosis [36]. Ferroptotic cancer cells release oncogenic KRAS protein which drives macrophages to enrich and switch to an M2-like pro-tumoral phenotype via STAT-dependent fatty acid oxidation [37, 38]. These TAMs exert higher resistance to ferroptosis compared with cancer cells. Moreover, erastin or SLC7A11 depletion to enhance ferroptosis were insufficient to induce macrophage ferroptosis [39, 40], implicating the considerable role of TAMs against T cell induced-anti-tumor response and the substantial benefit of depleting TAMs for T cell immunotherapy.

Depleting myeloid cell populations simultaneously could enhance anti-tumor efficacy of T cell immunotherapy even further, although complete depletion of myeloid cells could substantially increase clinical toxicities. Anti-GR1 antibody depletes both Ly6G^+^ granulocytes and Ly6C^hi^ monocytes simultaneously, while anti-CSF1R antibody depletes resident subset of monocytes and tissue- and tumor-associated macrophages together [41]. When tested their effect, anti-GR1 antibody depleted both granulocytes and monocytes, while anti-Ly6G antibody depleted just granulocytes. Though, anti-GR1 plus GD2-EATs did not produce a significant difference in TILs and in vivo anti-tumor response when compared with anti-Ly6G plus GD2-EATs. We also compared the efficacy of anti-CSF1R antibody with anti-Ly6C antibody or CL in neuroblastoma PDX model. Although anti-CSF1R antibody was more effective than anti-Ly6C antibody , it was not superior to CL. The effects of dual depletion of granulocyte and macrophage, or triple depletion of granulocyte, monocytes, and macrophages were not tested in this study, but deserve more in depth investigation.

One of the unexpected findings in our study is the effect of dexamethasone on TIM. Rather than reducing the potency, dexamethasone improved anti-tumor effects of BsAb treatment. The role of dexamethasone on TME and TAMs has not been fully investigated in the new era of T cell immunotherapies. Baseline use of corticosteroids was associated with poor outcome in patients with non-SCLC treated with PD-1 or PD-L1 blockades [42]. But interestingly, while the patients with poor-prognosis appeared to have worse outcome with the use of corticosteroids, those without a history of smoking or poor prognostic factors appeared to have better outcome [42, 43]. Dexamethasone premedication for CD19-BiTE (Blincyto®) did not impair its efficacy [44, 45]. Our study found that dexamethasone premedication did not induce apoptosis of CD8 T cells nor suppress the anti-tumor response of BsAb-driven T cell immunotherapy. On the contrary, it consistently and significantly improved in vivo anti-tumor efficacy of BsAb-driven T cell immunotherapy. Low-dose supplementary IL-2 inhibits dexamethasone-induced apoptosis in CD8 T cells by activating STAT-5 and altering cellular apoptotic potential [46, 47], and TAM suppression by dexamethasone [48] improved the antitumor immune response in cancer xenograft mouse models. Here we showed that dexamethasone depleted monocytes in the circulation and inhibited their recruitment into tumors, thereby decreasing TAMs and Ly6C^hi^ MDSCs, allowing cytotoxic T cells to successfully infiltrate and persist in solid tumors, resulting in significant improvements in anti-tumor response against neuroblastoma and osteosarcoma PDXs. Furthermore, dexamethasone regulates the expression of a huge number of genes in macrophages that are essential for their phagocytic activity, such as glucocorticoid-induced leucine zipper (Gilz) [49] and Annexin a1 [50–52]. More than 10,000 genomic glucocorticoid receptor (GR) binding sites are induced by dexamethasone in resting macrophages with more than 5,400 known GR target genes [53, 54]. Elucidating the effect of dexamethasone on transcriptomes of TAMs or TILs should yield a deeper insight into the role of glucocorticoid-mediated gene regulation on macrophage and T cell functions, and the mechanisms involved in enhanced anti-tumor efficacy of BsAb-T cell immunotherapies.

Previous studies have shown that tumor infiltrating myeloid cells (TIMs) including MDSCs and TAMs inhibit T cell infiltration into tumors and incapacitate TILs by accelerating T cell exhaustion and dysfunction [1]. The TIMs produce immunosuppressive factors or cytokines, induce Tregs, and drives CD8 T cell exhaustion by upregulation of PD-L1 [4, 55–57]. In this study, depleting TIMs increased TILs: macrophage depletion by anti-CSF1R antibody or CL significantly increased cytotoxic CD8 T cell infiltration. Previously, we showed that the PD-1 expression on CD3 T cells surged around day 16 after GD2-BsAb treatment was started [21], while the neuroblastoma PDXs harvested on day 10 post-treatment had few FoxP3^+^ Tregs irrespective of treatment (Additional file 1: Fig. S14A). When TILs were analyzed on day 60 post-treatment, myeloid cell depleting antibodies significantly increased frequencies of hCD8^+^ TILs, but the PD-1 expression on CD8^+^ TILs was not significantly different among groups (*P* = 0.45, Additional file 1: Fig. S14B). In this study, the expression of exhaustion markers with or without depleting antibodies was not investigated in detail. A full kinetic analysis of the exhaustion state of T cells following TIM depletion using extensive flow cytometry profiles and T cell RNA sequencing analysis should provide more insight and will be a subject of a future manuscript.

Despite these TIM depleting treatments, flow cytometry analysis of tumors exhibited a consistent presence of Ly6C^lo^ MDSCs in tumors. Ly6C^lo^ monocytes or Ly6C^lo^ MDSCs are known to express high levels of immunosuppressive proteins and soluble mediators, such as indoleamine 2,3-dioxygenase (IDO) and arginase, interfering CD8^+^ T cell proliferation and IFN-γ production [58, 59]. Although anti-Ly6C antibody did not induce a significant change, anti-Ly6G antibody, anti-CSF1R antibody, CL, and dexamethasone all increased Ly6C^lo^ MDSCs in tumors inversely to each depleted myeloid cell population. Macrophages and MDSCs are likely derived from CD11b^+^Ly6G^−^Ly6C^lo^ common myeloid progenitor (CMP) or granulocyte monocyte progenitor (GMP)-like precursors [60, 61], where M-MDSCs differentiate into PMN-MDSCs within the TME through the activity of IRF8 [61–64]. TIM depleting strategies could increase CD11b^+^Ly6G^−^Ly6C^lo^ precursor cell production, hence increasing Ly6C^lo^ MDSC. The biochemical and cellular effects of myeloid cell depleting strategies on MDSC and TAM differentiation are likely to be more complex and need to be investigated in depth. In addition, the effect of Ly6C^lo^ monocyte/MDSC targeting treatment such as liposomal doxorubicin [59] on T-BsAb based immunotherapy also needs to be tested.

Limitations of our preclinical model should also be noted. In our experiments, human cancer cell line or patient-derived tumors were passaged in immunodeficient BRG mice. The BsAbs were built for clinical application, specific for human antigens. We used human T cells as effector cells to study their infiltration into human tumor xenografts, whereas TIMs were mouse-derived. Previous studies of human and mouse TIMs have demonstrated how their subpopulation structures were conserved across patients and species, with a near-complete congruence between human and mouse for dendritic cells, monocytes and neutrophils, though not macrophages [65]. We are aware that tumors consist of many non-cancer cells including innate and adaptive immune cells, fibroblasts, endothelial cells, and lymphatics, which all interact to influence tumorigenesis [66, 67]. Given the species specificity of cell to cell, and cytokine or chemokine to receptor interactions, this preclinical model cannot fully represent the interactions between human tumor cells and human TME. Even after we ruled out the allogeneic effect between human T cells and xenografts from different donors [25], there are still the xenogeneic effect of human cells in a murine microenvironment. Although a fully syngeneic mouse model in an immunocompetent host can be used, there are substantial limitations: (1) we cannot study human T cells, (2) the tumors would no longer be human-derived, and (3) the therapeutics need to be reformatted to target mouse tumor cells, and they are highly immunogenic in immunocompetent mice with neutralizing antibodies developed within 2 weeks. In addition, (4) reformulating the therapeutics for a syngeneic immunocompetent mouse system (anti-tumor IgG and anti-mouse CD3) puts another major limitation on interpreting the results for their clinical application for humans, losing their clinical relevance.

## Conclusion

In conclusion, we demonstrated the importance of TIMs in suppressing BsAb-mediated intratumoral T cell infiltration and in vivo anti-tumor activity. We presented the individual contribution of PMN-MDSCs, M-MDSCs, and TAMs using specific myeloid cell depleting antibodies and inhibitors. BsAbs built on the IgG[L]-scFv platform can turn “cold” tumors into “hot” tumors by driving T cells into them. Myeloid cells following the TILs can interfere with T cell functions. Depletion of TIMs using specific antibodies or dexamethasone markedly enhanced the anti-tumor effects of BsAb-driven T cell immunotherapy by increasing intratumoral T cell infiltration and their persistence. TIM depleting strategies could improve the efficacy of BsAb-based T cell immunotherapy. These data support further investigation of the TME to identify the precise phenotypic and molecular profiles of TIMs to expand curative potential of T-BsAb based immunotherapies against solid tumors.

Bispecific antibodies built on the IgG[L]-scFv platform can turn “cold” tumors into “hot” tumors by driving T cells into them. Myeloid cells following the TILs can interfere with T cell functions. Depletion of TIMs using specific antibodies or dexamethasone markedly enhanced the anti-tumor effects of BsAb-driven T cell immunotherapy by increasing intratumoral T cell infiltration and their persistence. TIM depleting strategies could improve the efficacy of BsAb-based T cell immunotherapy.

## Supplementary Information

**Additional file 1. Supplementary Table S1.** Distribution of CD3, CD4, and CD8 T cells in neuroblastoma patient-derived xenografts by positive pixel count analyses. Abbreviations; CL, clodronate liposome. **Supplementary Table S2**. Tumor-infiltrating lymphocytes quantified by the area under each BLI curvesSupplementary Table S2. Tumor-infiltrating lymphocytes are quantified by the area under each BLI curve. Abbreviations; SCLC, small cell lung carcinoma; CDX, cell line-derived xenograft; PDX, patient-derived xenograft; CL, clodronate liposome; LD, low-dose; ID, intermediate-dose. **Supplementary Fig. S1**. (A) Neuroblastoma PDX bearing mice were treated by GD2-BsAb armed T cells (GD2-EATs) or unarmed T cells. (B) Tumors were harvested on day 10 and analyzed by flow cytometry. G-MDSC was gated as anti-mouse CD45+CD11b+Ly6G+; M-MDSC was gated as anti-mouse CD45+CD11b+Ly6G-Ly6Chi; TAM was gated as antimouse CD45+CD11b+Ly6G-Ly6CloF4/80+; Ly6Clo MDSC was gated as anti-mouse CD45+CD11b+Ly6GLy6CloF4/80-. The frequencies of each tumor-infiltrating leukocyte were compared among groups: a, no treatment; b, unarmed T cells; c, GD2-EATs. **Supplementary Fig. S2**. (A) Peripheral blood mononuclear cells (PBMCs) and GD2-BsAb were administered intravenously to the mice bearing 143B osteosarcoma cell line-derived xenograft (CDX). (B) Tumors were harvested on day 14 and immunohistochemical (IHC) stained with anti-CD11b antibody and anti-CD3 antibody. **Supplementary Fig. S3**. (A) Tumors were harvested on day 10 and analyzed by flow cytometry. G-MDSC was gated as anti-mouse CD45+CD11b+Ly6G+; M-MDSC was gated as anti-mouse CD45+CD11b+Ly6G-Ly6Chi; TAM was gated as antimouse CD45+CD11b+Ly6G-Ly6CloF4/80+; Ly6Clo MDSC was gated as anti-mouse CD45+CD11b+Ly6GLy6CloF4/80-. a, GD2-EATs; b, GD2-EATs plus anti-Ly6G antibody; c, GD2-EATs plus anti-Ly6C antibody; d, GD2-EATs plus anti-CSF1R antibody; e, GD2-EATs plus clodronate liposome (CL). **Supplementary Fig. S4**. (A) Osteosarcoma cell line xenografts were treated by GD2-BsAb armed T cells (GD2-EATs) or unarmed T cells with anti-Gr1 antibody or anti-Ly6G antibody. (B) CBC analyses were done on day 5 and compared among groups. (C) Tumors harvested on day 60 were analyzed by flow cytometry, and the frequencies of human CD45(+) or human CD8 (+) tumor-infiltrating lymphocytes were compared among groups. (D) Immunohistochemical (IHC) staining of tumor sections by anti-human CD3 antibody (x10) on day 60 post-treatment. The number of T cells was compared among groups using Q-path analysis. (E) In vivo anti-tumor effect of GD2-EATs with anti-Ly6G or anti-GR1 antibody was tested against 143B osteosarcoma cell line xenografts. **Supplementary Fig. S5**. (A) Luciferase transduced T cells [Luc(+) T cells] or Luciferase transduced GD2-BsAb armed T cells [Luc(+) GD2-EATs] were administered with anti-Ly6G antibody to the mice bearing neuroblastoma patient-derived xenograft (PDX). (B) Bioluminescence in the lesions of the tumor was monitored. The bioluminescence images on day 8 and quantification of the bioluminescence in the lesions of the tumor. **Supplementary Fig. S6**. (A) Osteosarcoma cell line xenografts were treated by GD2-BsAb armed T cells (GD2-EATs) with anti-Ly6C antibodies. (B) CBC analyses were done on day 5 and compared among groups. (C) Tumors harvested on day 60 were analyzed by flow cytometry, and the frequencies of human CD45(+) or human CD8 (+) tumor-infiltratinglymphocytes were compared among groups. (D) Immunohistochemical (IHC) staining of tumor sections by antihuman CD3 antibody (x10) on day 60 post-treatment. The number of T cells was compared among groups using Qpath analysis. (E) In vivo anti-tumor effect of GD2-EATs with anti-Ly6C antibody was tested against 143B osteosarcoma cell line xenografts. **Supplementary Fig. S7**. (A) Clodronate liposome (CL, 100μL) depleted macrophages in the liver. Livers were stained with immunofluorescence antibodies (green, murine CD68; blue, nuclei). (B) Anti-CSF1R antibody and 10μL of CL successfully depleted macrophages in the spleen. Spleens were processed with immunohistochemical (IHC) staining using murine CD68 antibody. (C) IHC staining of human CD3(+) T cells in 143B tumor sections aftertreatment with GD2-EATs plus anti-CSF1R antibody (x20). CD3(+) T cell number was compared with tumors treated with GD2-EATs alone. (D) IHC staining of human CD45(+) T cells in M14 tumor sections after treatment with T cells plus GD2-BsAb and CL (x10), and the number of CD45(+) T cells was compared. **Supplementary Fig. S8**. (A) Luciferase transduced T cells [Luc(+) T cells] were injected with clodronate liposome (CL) to small cell lung cancer (SCLC) cell line xenografts. The bioluminescence of Luc(+) T cell was monitored. The bioluminescence images on day 4 and quantitation of the bioluminescence in the lesions of the tumor. (B) Luciferase transduced HER2-BsAb armed T cells [Luc(+) HER2-EATs] were administered with anti-CSF1R antibody to HER2(+) osteosarcoma PDXs bearing mice. The bioluminescence of Luc(+) HER2-EATs was monitored. The bioluminescence images on day 4 and quantitation of the bioluminescence in the lesions of the tumor. **Supplementary Fig. S9**. (A) In vivo anti-tumor effect of GD2-BsAb armed T cells (GD2-EATs) plus clodronate liposome (CL) was studied in 143B osteosarcoma cell line xenograft model. (B) The anti-tumor effect of GD2-EATs plus anti-CSF1R antibody was tested against osteosarcoma patient-derived xenografts (PDXs), and overall survival was analyzed.**Supplementary Fig. S10**. (A) Increasing doses of dexamethasone with GD2-BsAb armed T cells (GD2-EATs) were administered to mice bearing neuroblastoma patient-derived xenograft (PDX). TH1 cell cytokines were measured at different time points post-GD2-BsAb armed T cell (GD2-EAT) injection. (B) In vivo TH1 cell cytokine release was compared amonggroups. **Supplementary Fig. S11**. (A) Tumors were harvested on day 25 post-treatment and analyzed by flow cytometry. G-MDSC was gated as antimouse CD45+CD11b+Ly6G+; M-MDSC was gated as anti-mouse CD45+CD11b+Ly6G-Ly6Chi; TAM was gated as anti-mouse CD45+CD11b+Ly6G-Ly6CloF4/80+; Ly6Clo MDSC was gated as anti-mouse CD45+CD11b+Ly6G-Ly6CloF4/80-. G1, no treatment; G2, unarmed T cells; G3, unarmed T cells plus high-dose (HD) dexamethasone;G4, GD2-EATs; G5, GD2-EATs plus low-dose (LD) dexamethasone; G6, GD2-EATs plus intermediate-dose (ID) dexamethasone; G7, GD2-EATs plus high-dose (HD) dexamethasone.**Supplementary Fig. S12**. (A) Luciferase transduced T cells [Luc(+) T cells] or Luciferase transduced GD2-BsAb armed T cells [Luc(+) GD2-EATs] were administered with increasing doses of dexamethasone to the mice bearing osteosarcoma patientderived xenograft (PDX). (B) Bioluminescence in the lungs and in the lesions of the tumor was monitored. Thebioluminescence images in the lungs on day 1 and in the lesions of tumor on day 5 and quantitation of the bioluminescence in the lesions of the tumor. **Supplementary Fig. S13**.(A) In vivo anti-tumor effect of dexamethasone premedication on GD2-BsAb directed T cell immunotherapy. 1x107 of T cells and 5μg of GD2-BsAb were administered with increasing doses of dexamethasone to mice bearing neuroblastoma patient-derived xenograft (PDX). (B) Increasing doses of dexamethasone with GD2-BsAb armed T cells (GD2-EATs) were administered to treat osteosarcoma PDXs. Tumor growth, body weight change, and overallsurvival were analyzed.

## Data Availability

All data generated or analyzed during this study are included in this published article or uploaded as supplementary information.

## Abbreviations

AUC: Area under curve
BLI: Bioluminescence intensity
BRG: BALB-Rag2-/-IL-2R-γc-KO
BsAb: Bispecific antibody
CAR: Chimeric antigen receptor
CDX: Cell line derived xenograft
CL: Clodronate liposome
CMP: Common myeloid progenitor
EAT: Ex vivo BsAb armed T cells
FBS: Fetal bovine serum
GMP: Granulocyte monocyte progenitor
IACUC: Institutional Animal Care and Use Committee
IDO: Indoleamine 2,3-dioxygenase
IF: Immunofluorescence
IHC: Immunohistochemistry
MDSC: Myeloid-derived suppressor cell
M-MDSC: Monocytic myeloid-derived suppressor cell
NO: Nitric oxide
PB: Peripheral blood
PBMC: Peripheral blood mononuclear cells
PDX: Patient-derived xenograft
PMN-MDSC: Polymorphonuclear myeloid-derived suppressor cell
SCLC: Small cell lung cancer
TAM: Tumor associated macrophage
TIL: Tumor infiltrating lymphocyte
TIM: Tumor infiltrating myeloid cells
TME: Tumor microenvironment
