# Supplementary material for: Modulating tumor infiltrating myeloid cells to enhance bispecific antibody-driven T cell infiltration and anti-tumor response
Source: J Hematol Oncol. 2021 Sep 8;14:142. doi: 10.1186/s13045-021-01156-5 (PMC8424962; doi:10.1186/s13045-021-01156-5)

Modulating Tumor Infiltrating Myeloid Cells to Enhance Bispecific Antibody-driven T cell Infiltration and Anti-tumor Response

Jeong A Park, M.D., Ph. D.[1,2], Linlin Wang, Ph.D.[1,2] and Nai-Kong V. Cheung, M.D., Ph.D.[1]*

[1]Department of Pediatrics, Memorial Sloan Kettering Cancer Center, New York, NY

[2] Co first authors

Running title: Modulating myeloid cells to enhance T-cell bispecific antibody

Kew Words: Bispecific antibody; Dexamethasone; Disialogangliosides; Ex vivo Bispecific Antibody Armed T-cells (EATs); human epidermal growth factor receptor 2 (HER2); Immunotherapy; Myeloid-derived suppressor cell; T cell; Tumor infiltrating myeloid cell; Tumor microenvironment; Tumor-associated macrophage

*Corresponding author:

Nai-Kong V. Cheung, M.D., Ph.D.

Department of Pediatrics

Memorial Sloan Kettering Cancer Center

1275 York Avenue

New York, NY 10065.

Phone: +1 646-888-2313

Email: cheungn@mskcc.org

Supplementary Table S1. Distribution of CD3, CD4, and CD8 T cells in neuroblastoma patient-derived xenografts by positive pixel count analyses .

Treatment	CD3 (% positive cells ± SD)	*P*-value (vs.GD2-EATs)	CD4 (% positive cells ± SD)	*P*-value (vs. GD2-EATs)	CD8 (% positive cells ± SD)	*P*-value (vs.GD2-EATs)	Ratio of % CD8 to % CD4 T cells	*P*-value (vs.GD2-EATs)
Unarmed T cells	1.4 ± 0.7	0.001	0.8 ± 0.4	0.005	0.07 ± 0.06	0.001	0.1 ± 0.07	0.03
GD2-EATs	15 ± 5	-	5.0 ± 2	-	1.1 ± 0.3	-	0.2 ± 0.06	-
GD2-EATs + anti-Ly6G	57 ± 19	0.005	28 ± 15	0.02	5.0 ± 3	0.03	0.2 ± 0.04	0.28
GD2-EATs + anti-Ly6C	35 ± 12	0.02	15.5 ± 10	0.08	4.4 ± 4	0.15	0.3 ± 0.08	0.81
GD2-EATs + anti-CSF1R	71 ± 29	0.009	26 ± 14	0.03	19 ± 12	0.025	0.8 ± 0.20	0.001
GD2-EATs + CL	66 ± 9	<0.0001	28 ± 12	0.01	16 ± 8	0.007	0.6 ± 0.04	0.0001

Abbreviations; CL, clodronate liposome

Supplementary Table S2. Tumor infiltrating lymphocytes quantified by area under each BLI curves

Control	TIM depletion treatment	Fold increase in TIL (AUC-BLI)	*P*-value	Tumor model	Figure
GD2-BsAb	GD2-EATs + anti-GR1	5.8	0.03	SCLC CDX	Fig.2B
GD2-BsAb	GD2-BsAb + anti-Ly6G	2.5	0.01	SCLC CDX	Fig.2B
GD2-EATs	GD2-EATs+ anti-Ly6G	2.9	0.01	neuroblastoma PDX	Fig.S5
GD2-EATs	GD2-EATs + anti-Ly6C	3.5	0.01	neuroblastoma PDX	Fig.4B
GD2-EATs	GD2-EATs + anti-CSF1R	6.6	0.08	neuroblastoma PDX	Fig.6B
HER2-EATs	HER2-EATs + anti-CSF1R	3.6	0.12	osteosarcoma PDX	Fig.S8B
GD2-EATs	GD2-EATs + CL	3.2	0.15	neuroblastoma PDX	Fig.6B
GD2-BsAb	GD2-BsAb + CL	4.5	0.07	SCLC CDX	Fig.S8A
GD2-EATs	GD2-EATs + LD dexamethasone	7.7	0.07	osteosarcoma PDX	Fig.8B
GD2-EATs	GD2-EATs + ID dexamethasone	14.0	0.04	osteosarcoma PDX	Fig.8B

Abbreviations; SCLC, small cell lung carcinoma; CDX, cell line-derived xenograft; PDX, patient-derived xenograft; CL, clodronate liposome; LD, low-dose; ID, intermediate-dose.

Supplementary Fig. S1.

(A) Neuroblastoma PDX bearing mice were treated by GD2-BsAb armed T cells (GD2-EATs) or unarmed T cells. (B) Tumors were harvested on day 10 and analyzed by flow cytometry. G-MDSC was gated as anti-mouse $CD45^{+}CD11b^{+}Ly6G^{+}$; M-MDSC was gated as anti-mouse $CD45^{+}CD11b^{+}Ly6G^{-}Ly6C^{hi}$; TAM was gated as anti-mouse $CD45^{+}CD11b^{+}Ly6G^{-}Ly6C^{lo}F4/80^{+}$; $Ly6C^{lo}$ MDSC was gated as anti-mouse $CD45^{+}CD11b^{+}Ly6G^{-}Ly6C^{lo}F4/80^{-}$. The frequencies of each tumor infiltrating leukocytes were compared among groups: a, no treatment; b, unarmed T cells; c, GD2-EATs.

Supplementary Fig. S2.

(A) Peripheral blood mononuclear cells (PBMCs) and GD2-BsAb were administered intravenously to the mice bearing 143B osteosarcoma cell line-derived xenograft (CDX). (B) Tumors were harvested on day 14 and immunohistochemical (IHC) stained with anti-CD11b antibody and anti-CD3 antibody.

Supplementary Fig. S3.

(A) Tumors were harvested on day 10 and analyzed by flow cytometry. G-MDSC was gated as anti-mouse $CD45^{+}CD11b^{+}Ly6G^{+}$; M-MDSC was gated as anti-mouse $CD45^{+}CD11b^{+}Ly6G^{-}Ly6C^{hi}$; TAM was gated as anti-mouse $CD45^{+}CD11b^{+}Ly6G^{-}Ly6C^{lo}F4/80^{+}$; $Ly6C^{lo}$ MDSC was gated as anti-mouse $CD45^{+}CD11b^{+}Ly6G^{-}Ly6C^{lo}F4/80^{-}$. a, GD2-EATs; b, GD2-EATs plus anti-Ly6G antibody; c, GD2-EATs plus anti-Ly6C antibody; d, GD2-EATs plus anti-CSF1R antibody; e, GD2-EATs plus clodronate liposome (CL).

Supplementary Fig. S4.

(A) Osteosarcoma cell line xenografts were treated by GD2-BsAb armed T cells (GD2-EATs) or unarmed T cells with anti-Gr1 antibody or anti-Ly6G antibody. (B) CBC analyses were done on day 5 and compared among groups. (C) Tumors harvested on day 60 were analyzed by flow cytometry, and the frequencies of human CD45(+) or human CD8 (+) tumor infiltrating lymphocyte were compared among groups. (D) Immunohistochemical (IHC) staining of tumor sections by anti-human CD3 antibody (x10) on day 60 post-treatment. The number of T cell was compared among groups using Q-path analysis. (E) In vivo anti-tumor effect of GD2-EATs with anti-Ly6G or anti-GR1 antibody was tested against 143B osteosarcoma cell line xenografts.

Supplementary Fig. S5.

(A) Luciferase transduced T cells [Luc(+) T cells] or Luciferase transduced GD2-BsAb armed T cells [Luc(+) GD2-EATs] were administered with anti-Ly6G antibody to the mice bearing neuroblastoma patient-derived xenograft (PDX). (B) Bioluminescence in the lesions of tumor was monitored. The bioluminescence images on day 8 and quantification of the bioluminescence in the lesions of tumor.

Supplementary Fig. S6.

(A) Osteosarcoma cell line xenografts were treated by GD2-BsAb armed T cells (GD2-EATs) with anti-Ly6C antibody. (B) CBC analyses were done on day 5 and compared among groups. (C) Tumors harvested on day 60 were analyzed by flow cytometry, and the frequencies of human CD45(+) or human CD8 (+) tumor infiltrating lymphocyte were compared among groups. (D) Immunohistochemical (IHC) staining of tumor sections by anti-human CD3 antibody (x10) on day 60 post-treatment. The number of T cell was compared among groups using Q-path analysis. (E) In vivo anti-tumor effect of GD2-EATs with anti-Ly6C antibody was tested against 143B osteosarcoma cell line xenografts.

Supplementary Fig. S7.

(A) Clodronate liposome (CL, 100μL) depleted macrophages in the liver. Livers were stained with immunofluorescence antibodies (green, murine CD68; blue, nuclei). (B) Anti-CSF1R antibody and 10μL of CL successfully depleted macrophages in the spleen. Spleens were processed with immunohistochemical (IHC) staining using murine CD68 antibody. (C) IHC straining of human CD3(+) T cells in 143B tumor sections after treatment with GD2-EATs plus anti-CSF1R antibody (x20). CD3(+) T cell number was compared with tumors treated with GD2-EATs alone. (D) IHC staining of human CD45(+) T cells in M14 tumor sections after treatment with T cells plus GD2-BsAb and CL (x10), and the number of CD45(+) T cell was compared.

Supplementary Fig. S8.

(A) Luciferase transduced T cells [Luc(+) T cells] were injected with clodronate liposome (CL) to small cell lung cancer (SCLC) cell line xenografts. The bioluminescence of Luc(+) T cell was monitored. The bioluminescence images on day 4 and quantitation of the bioluminescence in the lesions of tumor. (B) Luciferase transduced HER2-BsAb armed T cells [Luc(+) HER2-EATs] were administered with anti-CSF1R antibody to HER2(+) osteosarcoma PDXs bearing mice. The bioluminescence of Luc(+) HER2-EATs was monitored. The bioluminescence images on day 4 and quantitation of the bioluminescence in the lesions of tumor.

Supplementary Fig. S9.

(A) In vivo anti-tumor effect of GD2-BsAb armed T cells (GD2-EATs) plus clodronate liposome (CL) was studied in 143B osteosarcoma cell line xenograft model. (B) Anti-tumor effect of GD2-EATs plus anti-CSF1R antibody was tested against osteosarcoma patient-derived xenografts (PDXs), and overall survival was analyzed.

Supplementary Fig. S10.

(A) Increasing doses of dexamethasone with GD2-BsAb armed T cells (GD2-EATs) were administered to mice bearing neuroblastoma patient-derived xenograft (PDX). TH1 cell cytokines were measured at different time points post GD2-BsAb armed T cell (GD2-EAT) injection. (B) In vivo TH1 cell cytokine release was compared among groups.

Supplementary Fig. S11.

(A) Tumors were harvested on day 25 post-treatment and analyzed by flow cytometry. G-MDSC was gated as anti-mouse $CD45^{+}CD11b^{+}Ly6G^{+}$; M-MDSC was gated as anti-mouse $CD45^{+}CD11b^{+}Ly6G^{-}Ly6C^{hi}$; TAM was gated as anti-mouse $CD45^{+}CD11b^{+}Ly6G^{-}Ly6C^{lo}F4/80^{+}$; Ly6Clo MDSC was gated as anti-mouse $CD45^{+}CD11b^{+}Ly6G^{-}Ly6C^{lo}F4/80^{-}$. G1, no treatment; G2, unarmed T cells; G3, unarmed T cells plus high-dose (HD) dexamethasone; G4, GD2-EATs; G5, GD2-EATs plus low-dose (LD) dexamethasone; G6, GD2-EATs plus intermediate-dose (ID) dexamethasone; G7, GD2-EATs plus high-dose (HD) dexamethasone.

Supplementary Fig. S12.

(A) Luciferase transduced T cells [Luc(+) T cells] or Luciferase transduced GD2-BsAb armed T cells [Luc(+) GD2-EATs] were administered with increasing doses of dexamethasone to the mice bearing osteosarcoma patient-derived xenograft (PDX). (B) Bioluminescence in the lungs and in the lesions of tumor was monitored. The bioluminescence images in the lungs on day 1 and in the lesions of tumor on day 5 and quantitation of the bioluminescence in the lesions of tumor.

Supplementary Fig. S13.

(A) *In vivo* anti-tumor effect of dexamethasone premedication on GD2-BsAb directed T cell immunotherapy. $1x10^7$ of T cells and 5μg of GD2-BsAb were administered with increasing doses of dexamethasone to mice bearing neuroblastoma patient-derived xenograft (PDX). (B) Increasing doses of dexamethasone with GD2-BsAb armed T cells (GD2-EATs) were administered to treat osteosarcoma PDXs. Tumor growth, body weight change, and overall survival was analyzed.

Supplementary Fig. S14.

(A) Immunohistochemical (IHC) staining of tumor sections by anti-human FoxP3 antibody (x10). Neuroblastoma PDXs were harvested on day 10 post-treatment. a, no treatment; b, unarmed T cells; c, GD2-EATs; d, GD2-EATs plus anti-Ly6G antibody; e, GD2-EATs plus anti-Ly6C antibody; f, GD2-EATs plus anti-CSF1R antibody; g, GD2-EATs plus clodronate liposome (CL). (B) Osteosarcoma cell line 143B xenografts were treated by GD2-EATs with TIM depleting antibodies. Tumors harvested on day 60 were analyzed by flow cytometry, and the frequencies of human PD-1 positivity in human CD8+ TILs were compared among groups.

Supplementary Fig S1.

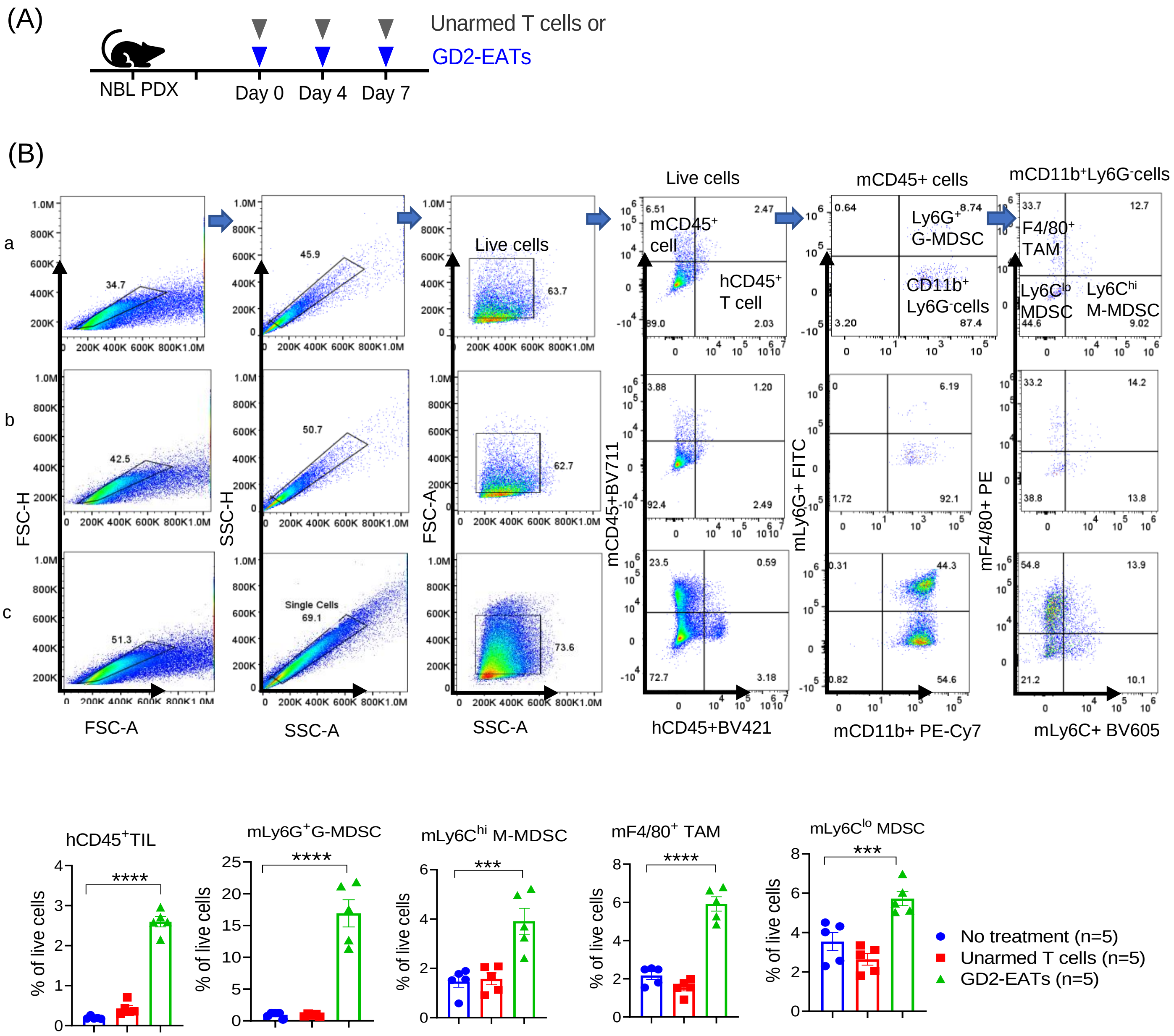

Supplementary Fig S2.

(A)

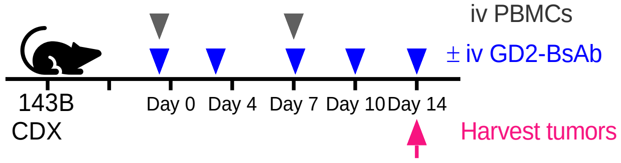

(B)

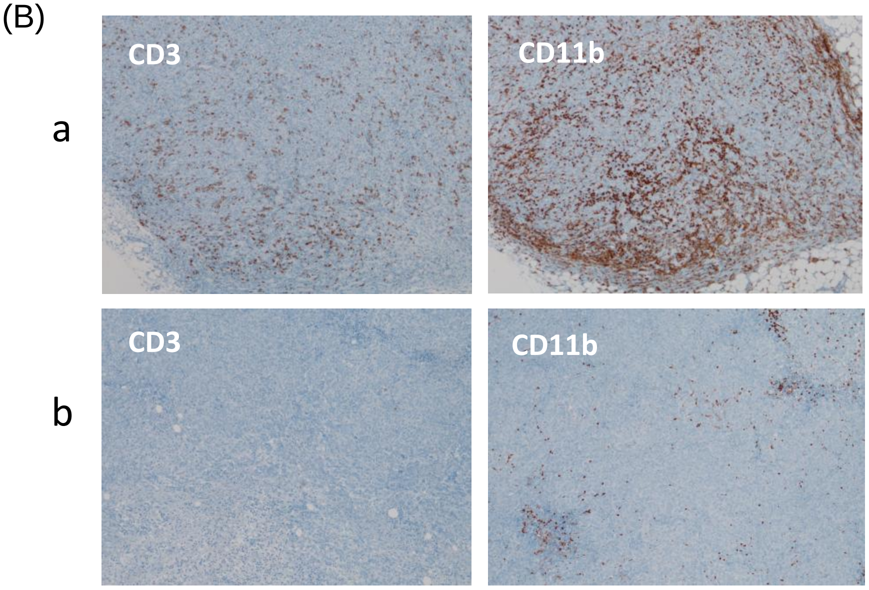

Supplementary Fig S3.

(A)

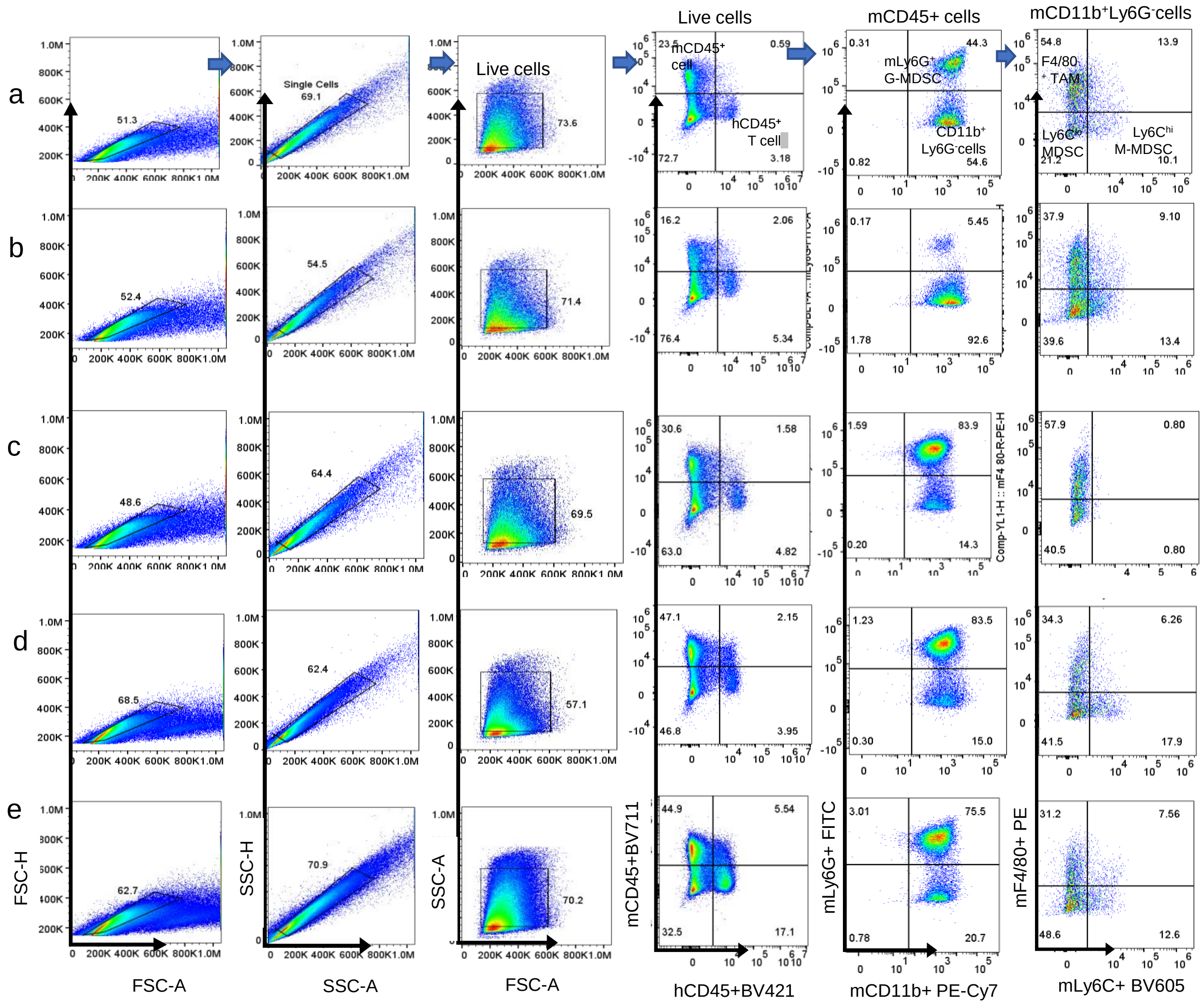

Supplementary Fig S4.

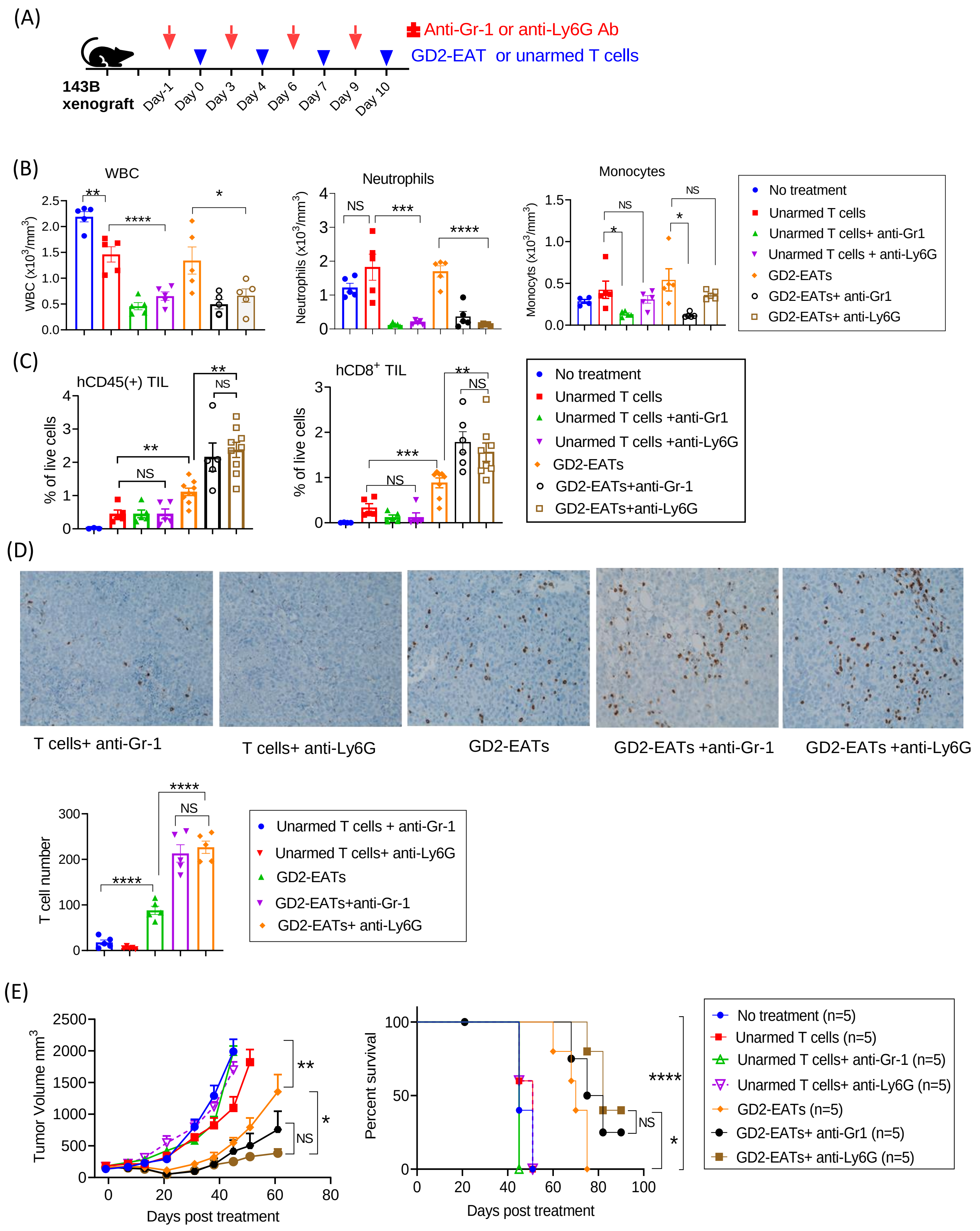

Supplementary Fig S5.

(A)

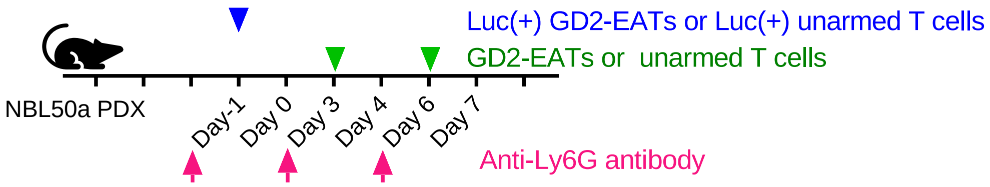

(B)

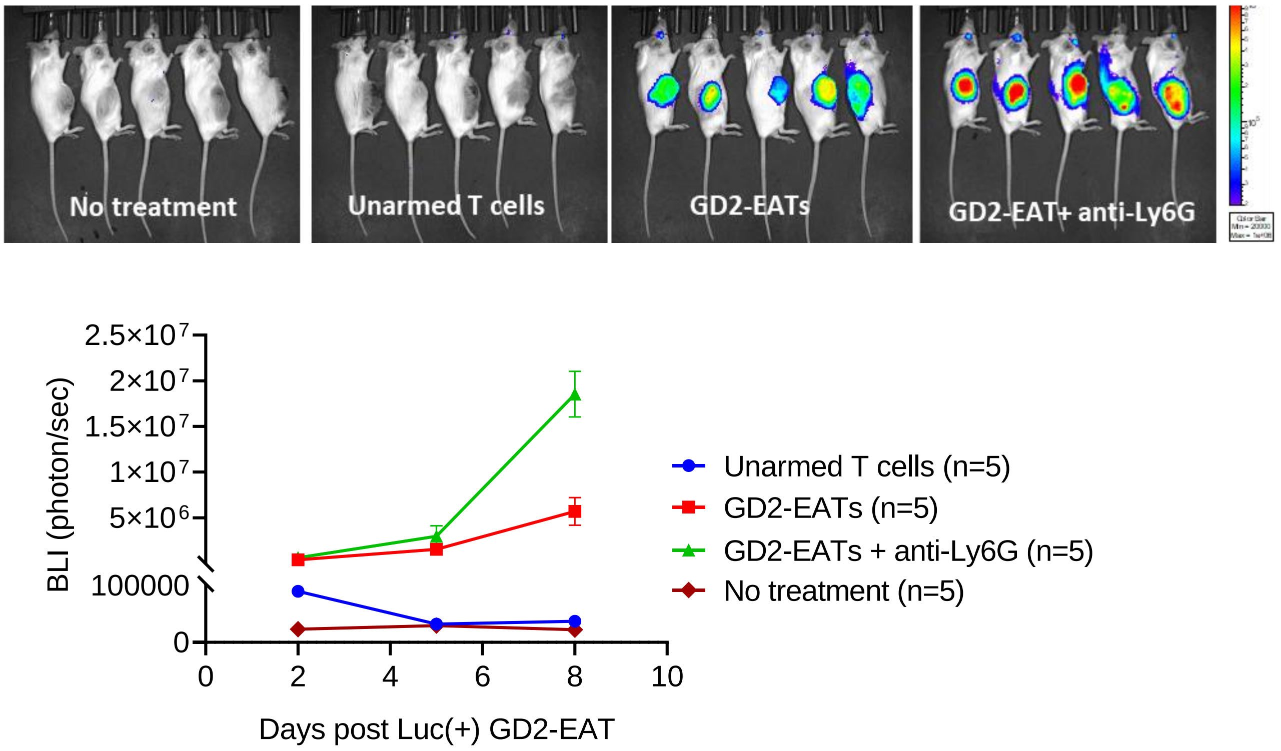

Supplementary Fig S6.

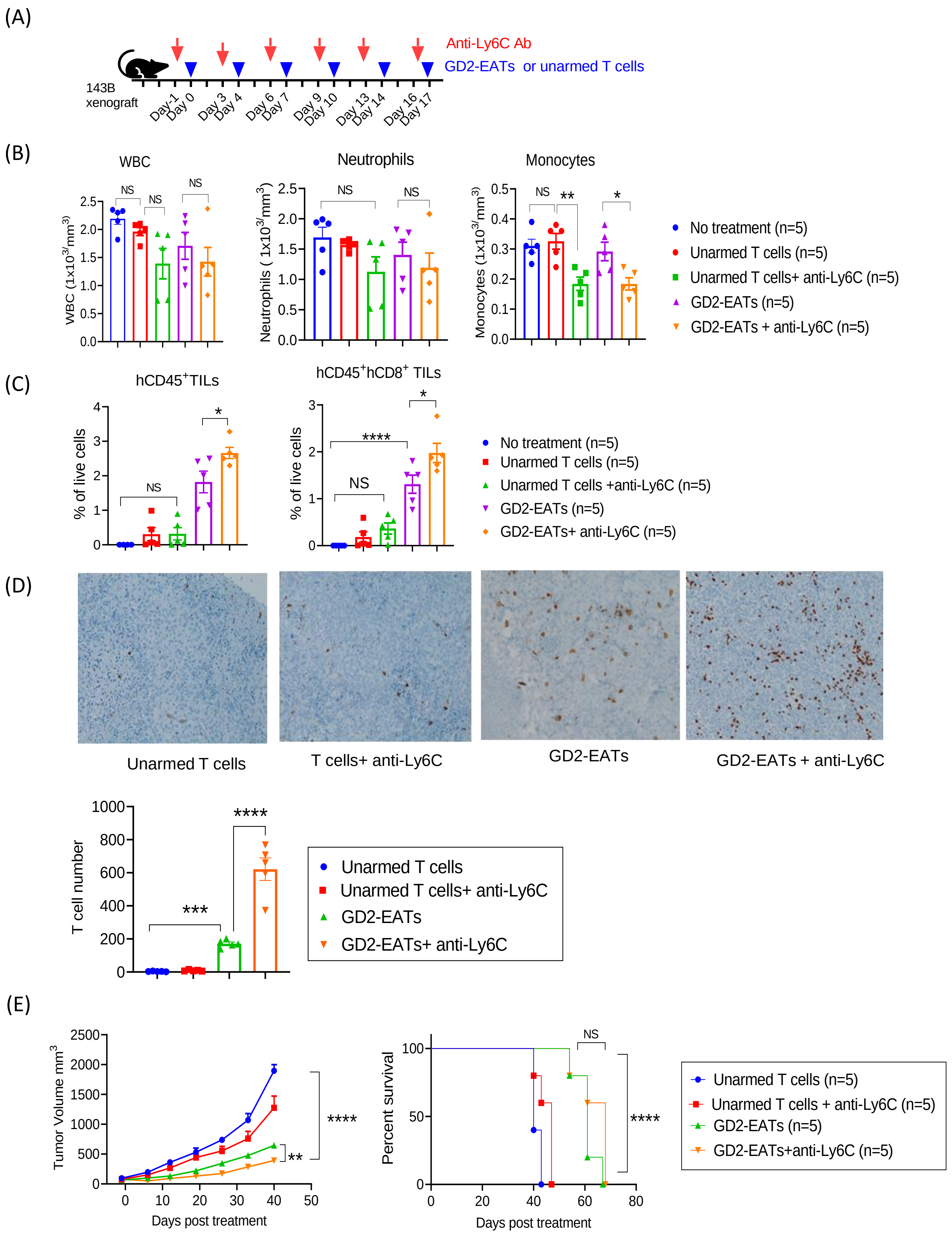

Supplementary Fig S7.

(A)

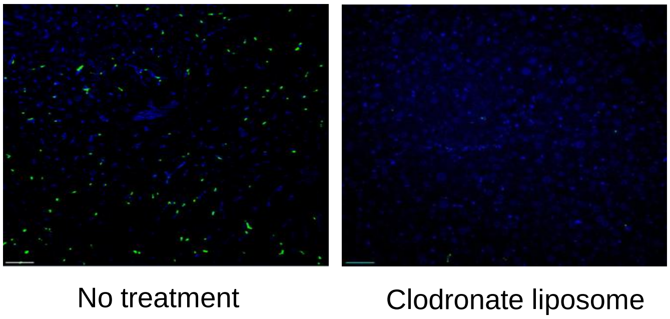

(B)

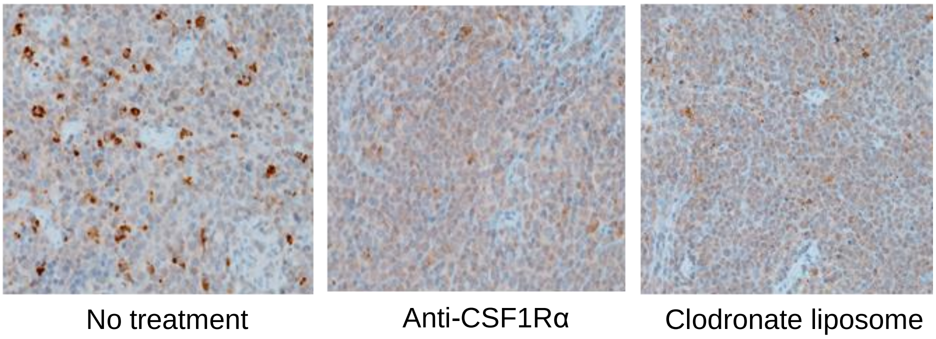

(C)

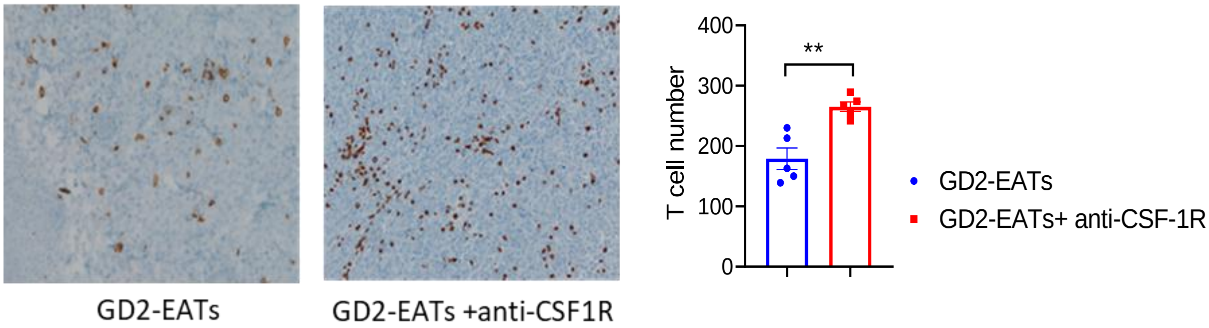

(D)

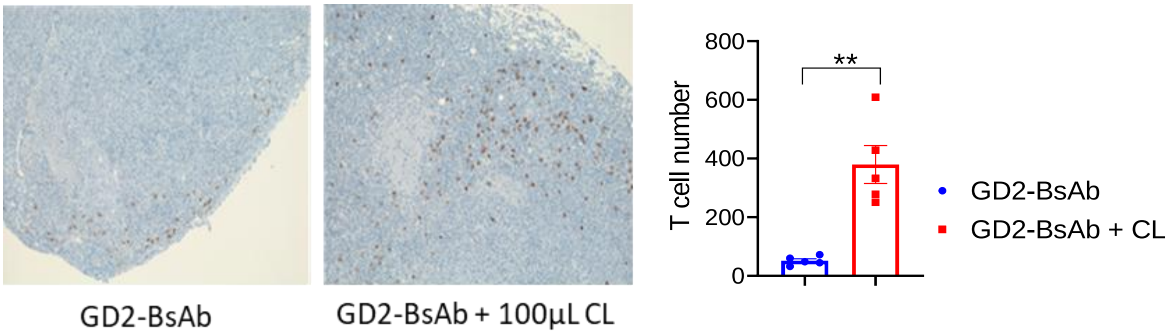

Supplementary Fig S8.

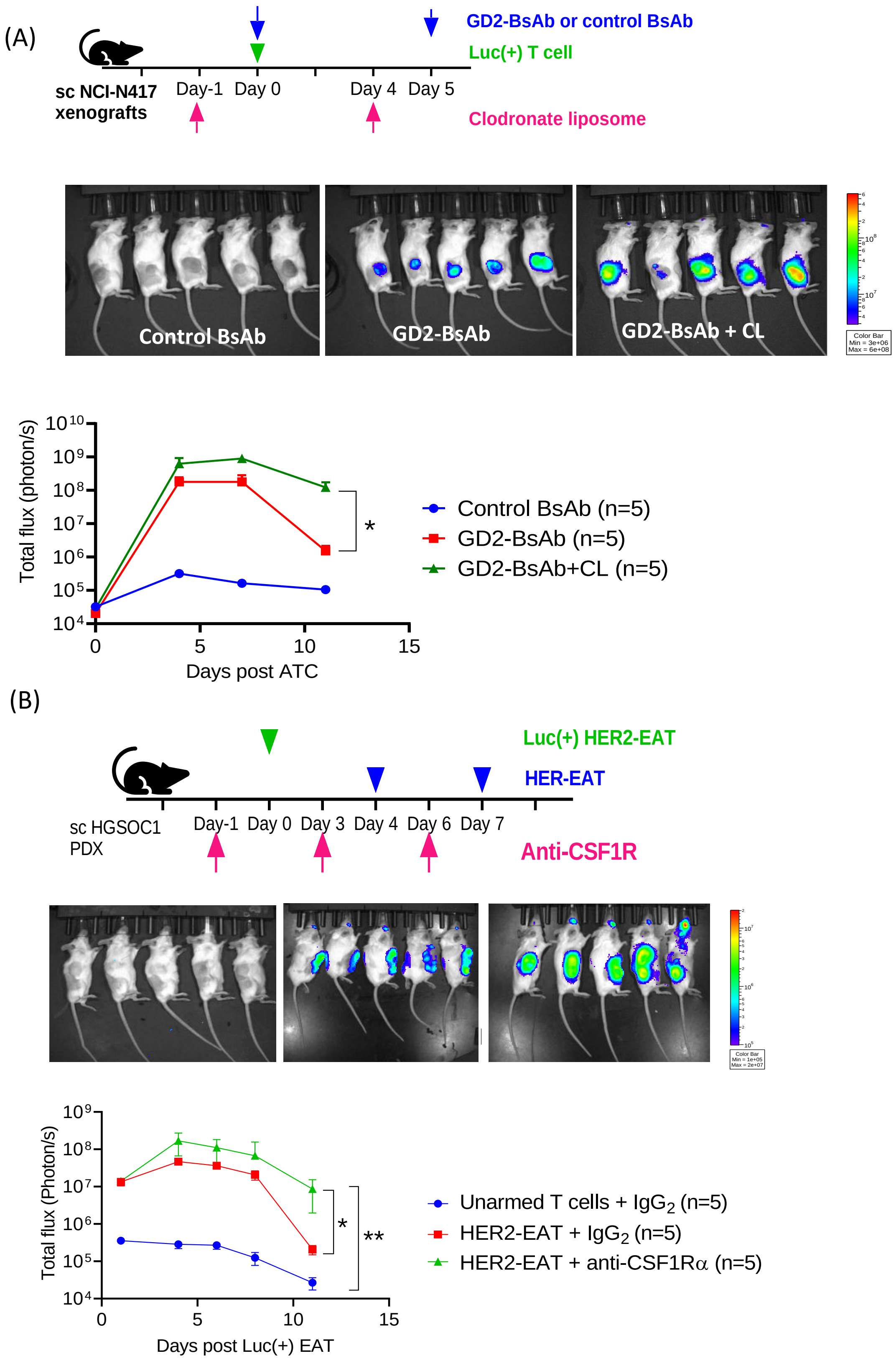

Supplementary Fig S9.

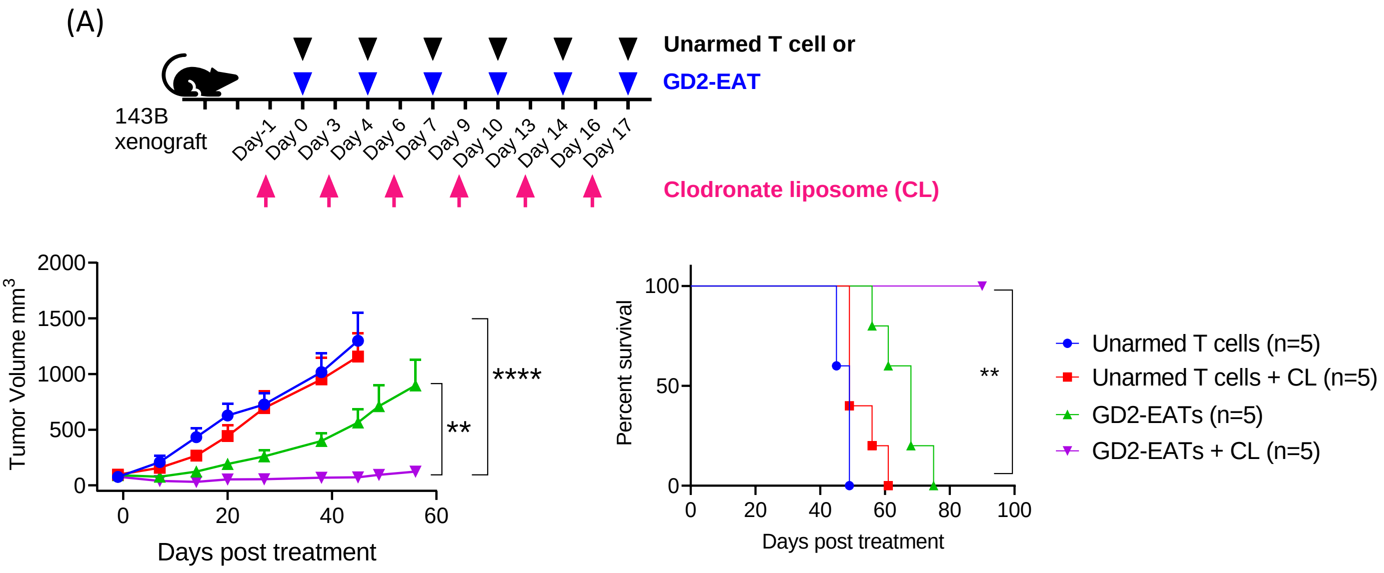

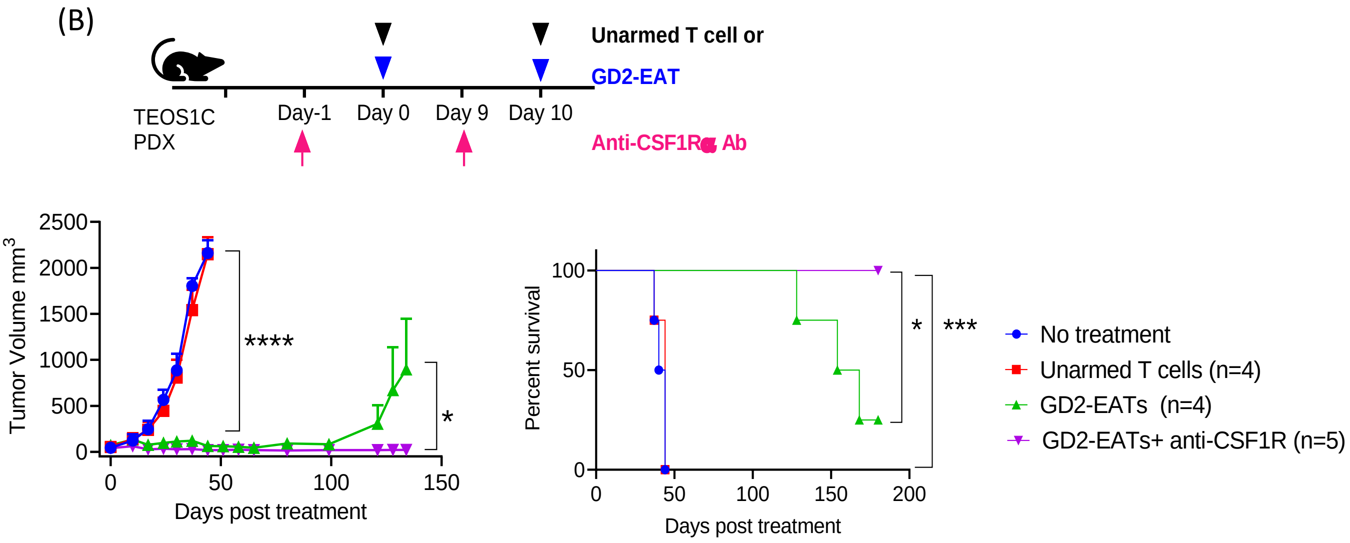

Supplementary Fig S10.

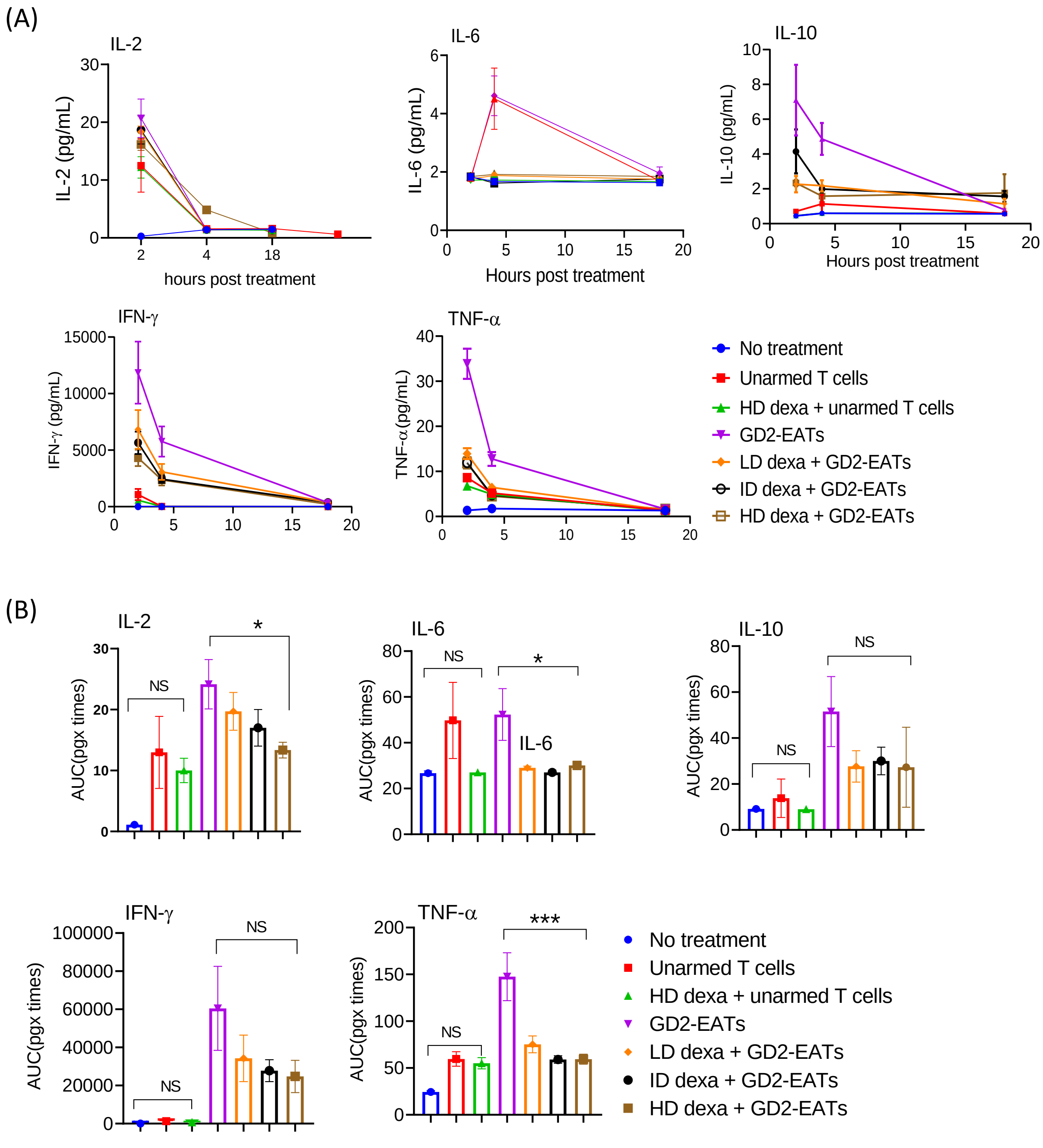

Supplementary Fig S11.

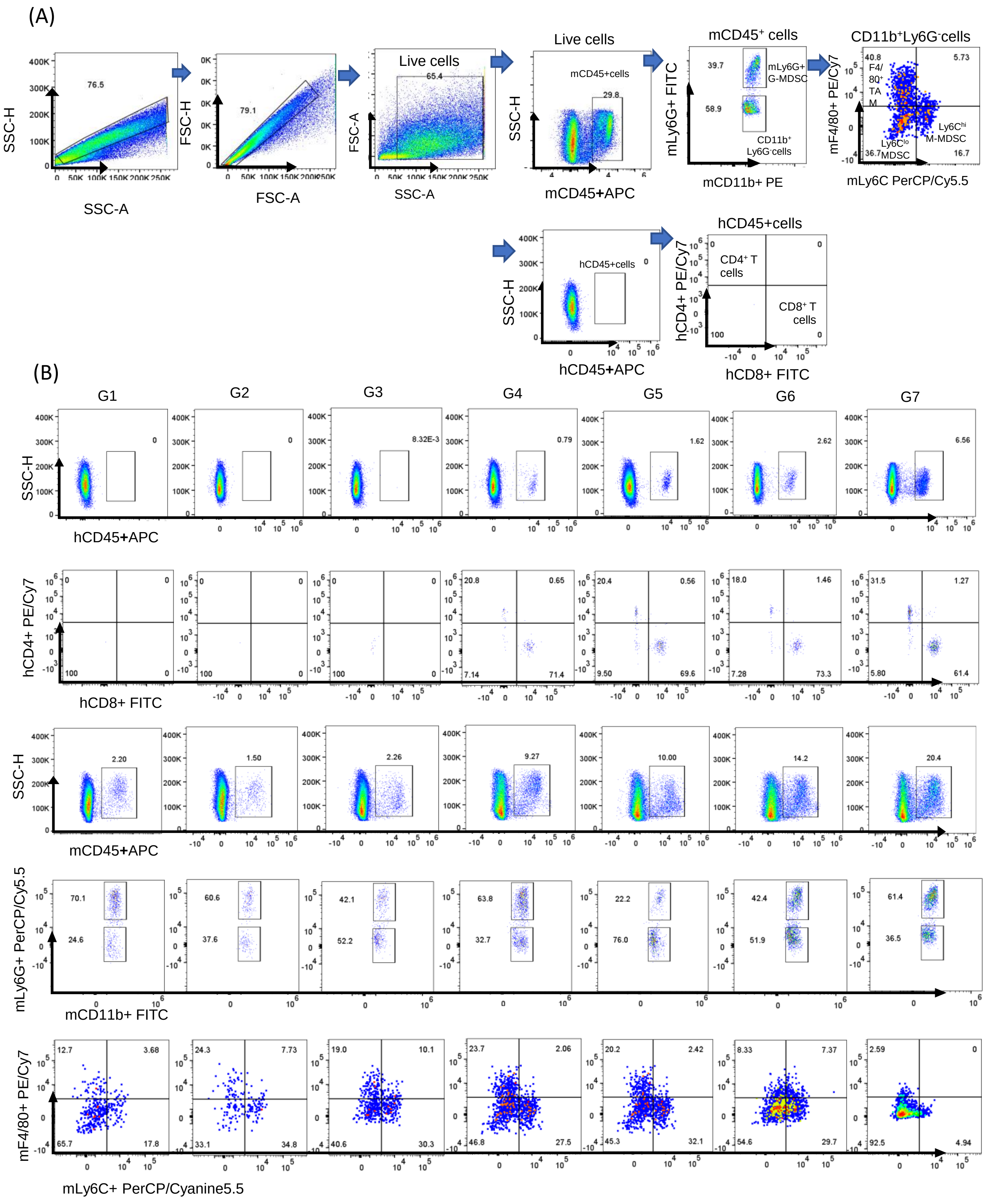

Supplementary Fig. S12

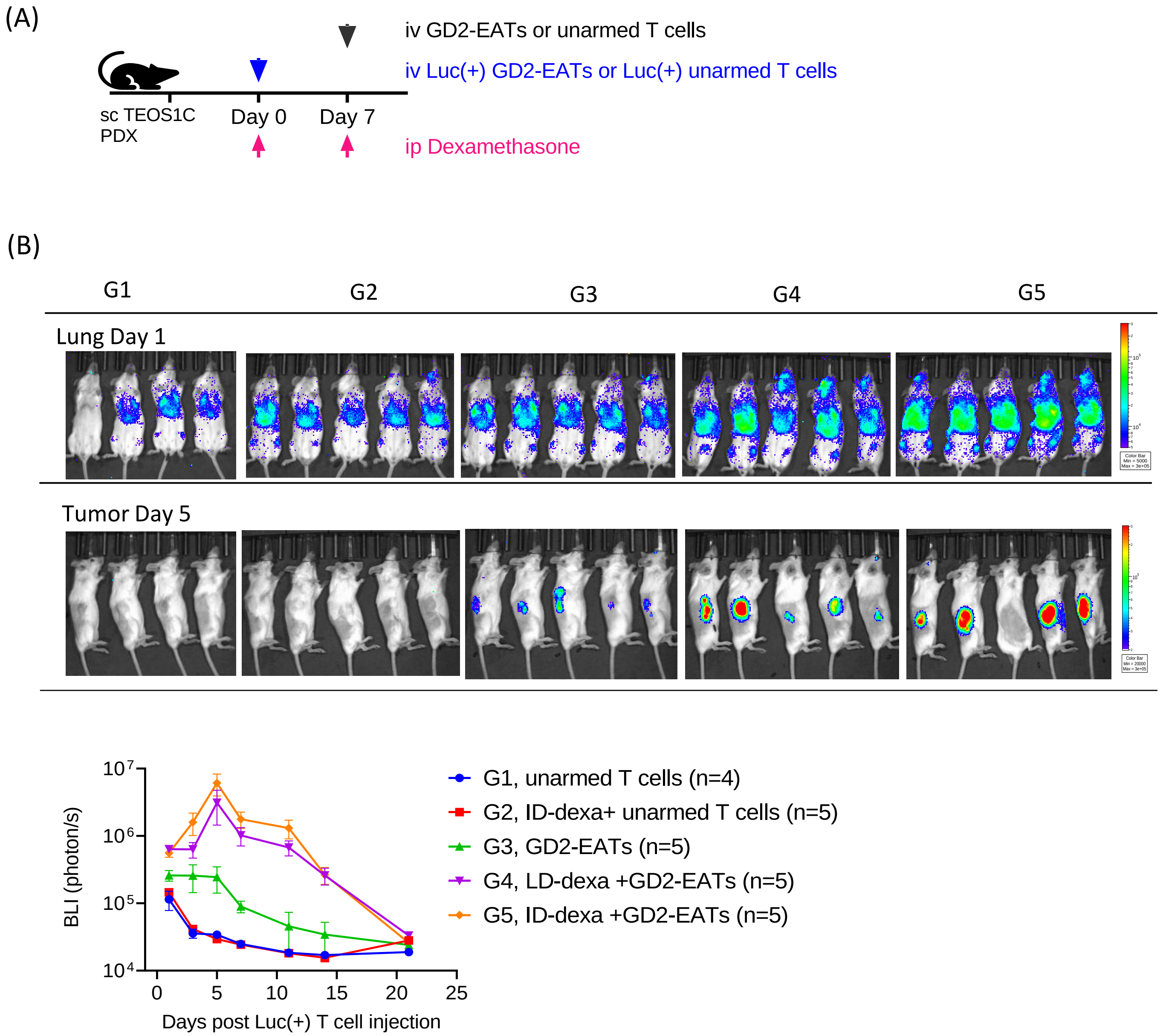

Supplementary Fig. S13.

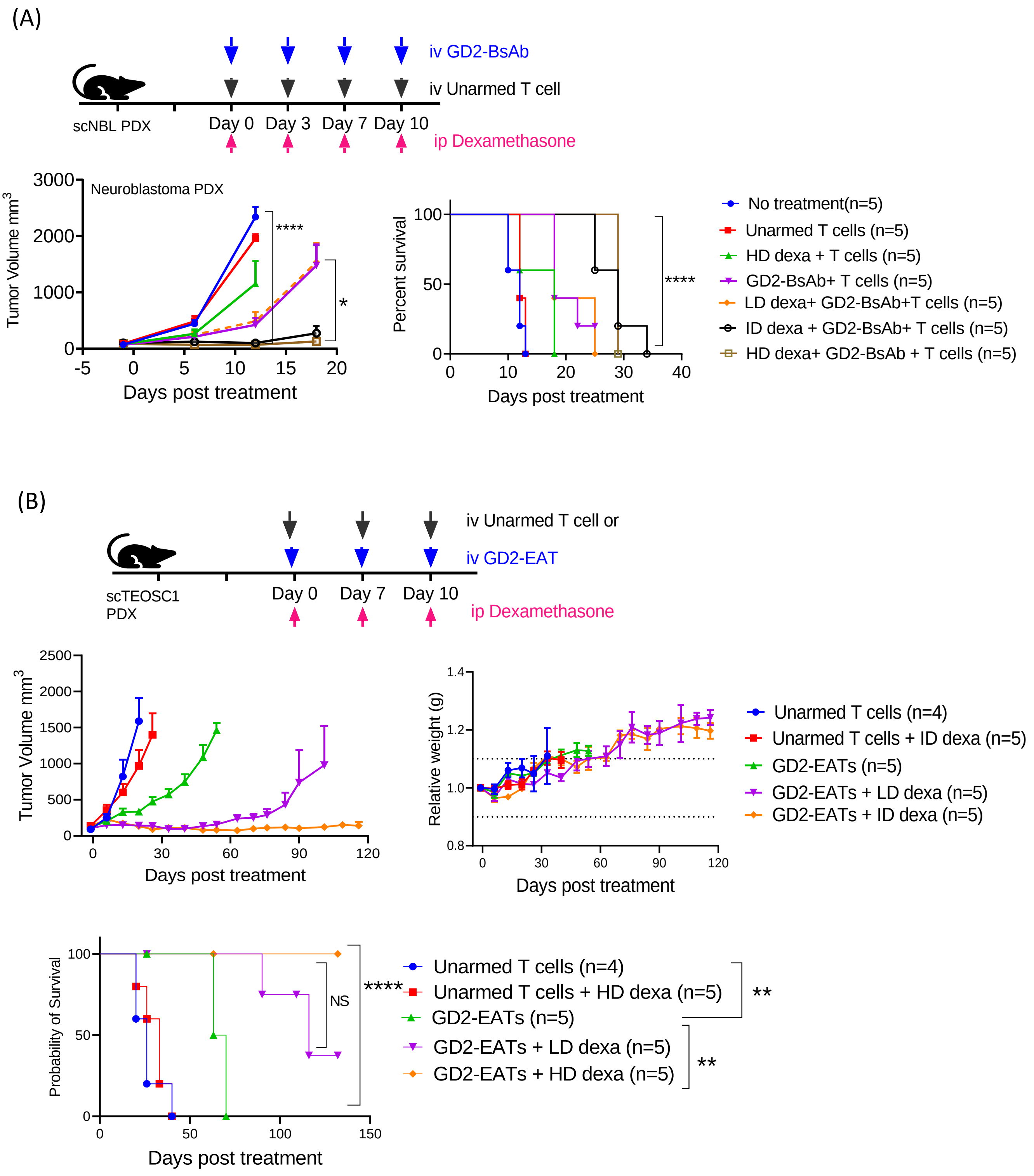

Supplementary Fig. S14.

(A)

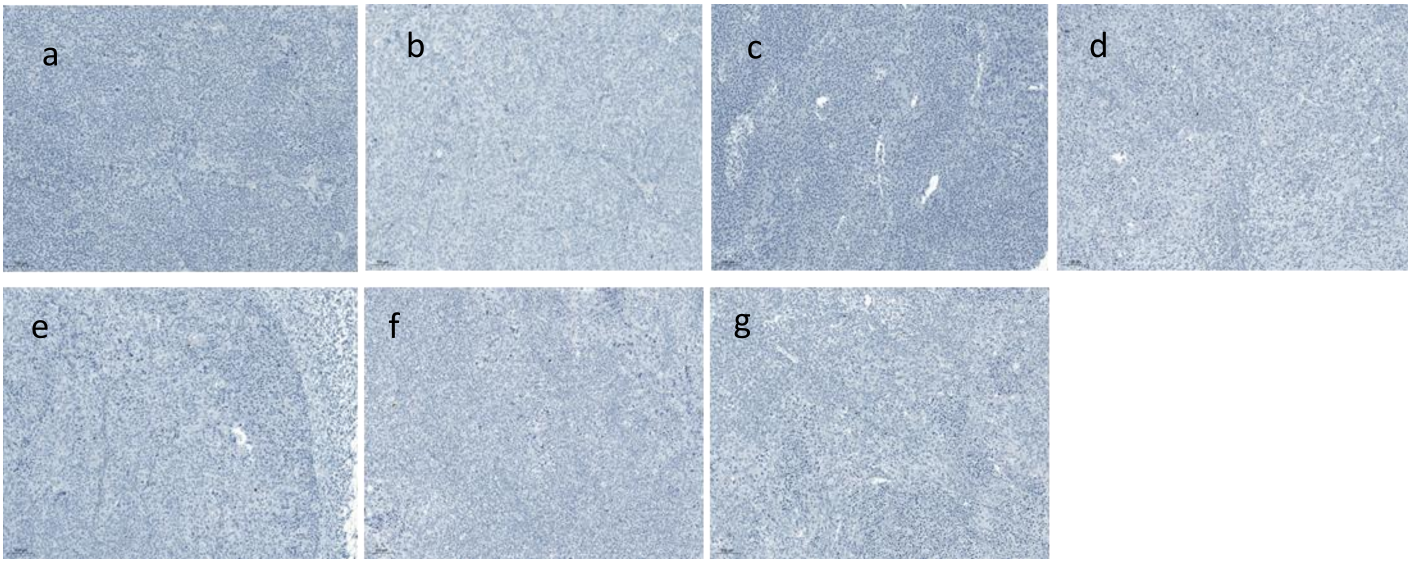

(B)

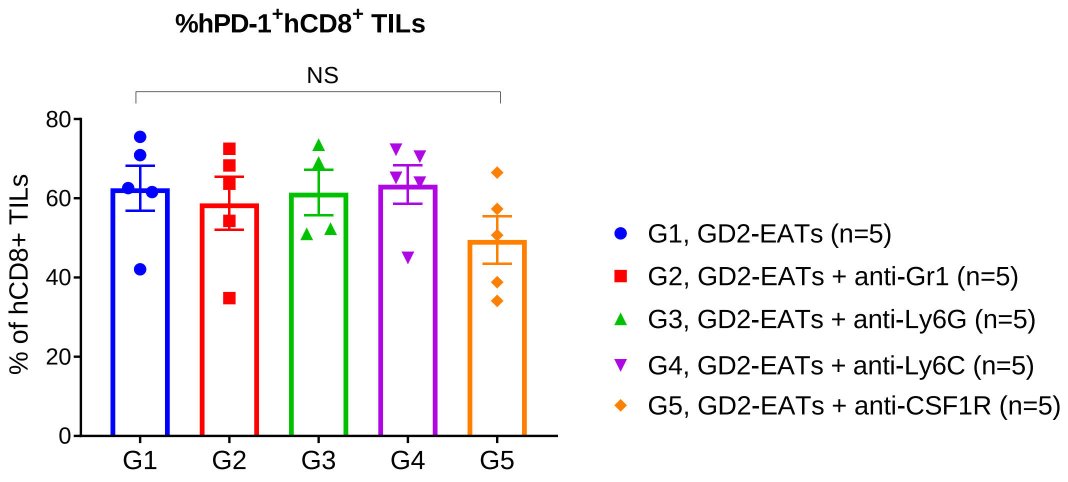

Supplement: Supplementary file 1 — Additional file 1. Supplementary Table S1. Distribution of CD3, CD4, and CD8 T cells in neuroblastoma patient-derived xenografts by positive pixel count analyses. Abbreviations; CL, clodronate liposome. Supplementary Table S2. Tumor-infiltrating lymphocytes quantified by the area under each BLI curvesSupplementary Table S2. Tumor-infiltrating lymphocytes are quantified by the area under each BLI curve. Abbreviations; SCLC, small cell lung carcinoma; CDX, cell line-derived xenograft; PDX, patient-derived xenograft; CL, clodronate liposome; LD, low-dose; ID, intermediate-dose. Supplementary Fig. S1. (A) Neuroblastoma PDX bearing mice were treated by GD2-BsAb armed T cells (GD2-EATs) or unarmed T cells. (B) Tumors were harvested on day 10 and analyzed by flow cytometry. G-MDSC was gated as anti-mouse CD45+CD11b+Ly6G+; M-MDSC was gated as anti-mouse CD45+CD11b+Ly6G-Ly6Chi; TAM was gated as antimouse CD45+CD11b+Ly6G-Ly6CloF4/80+; Ly6Clo MDSC was gated as anti-mouse CD45+CD11b+Ly6GLy6CloF4/80-. The frequencies of each tumor-infiltrating leukocyte were compared among groups: a, no treatment; b, unarmed T cells; c, GD2-EATs. Supplementary Fig. S2. (A) Peripheral blood mononuclear cells (PBMCs) and GD2-BsAb were administered intravenously to the mice bearing 143B osteosarcoma cell line-derived xenograft (CDX). (B) Tumors were harvested on day 14 and immunohistochemical (IHC) stained with anti-CD11b antibody and anti-CD3 antibody. Supplementary Fig. S3. (A) Tumors were harvested on day 10 and analyzed by flow cytometry. G-MDSC was gated as anti-mouse CD45+CD11b+Ly6G+; M-MDSC was gated as anti-mouse CD45+CD11b+Ly6G-Ly6Chi; TAM was gated as antimouse CD45+CD11b+Ly6G-Ly6CloF4/80+; Ly6Clo MDSC was gated as anti-mouse CD45+CD11b+Ly6GLy6CloF4/80-. a, GD2-EATs; b, GD2-EATs plus anti-Ly6G antibody; c, GD2-EATs plus anti-Ly6C antibody; d, GD2-EATs plus anti-CSF1R antibody; e, GD2-EATs plus clodronate liposome (CL). Supplementary Fig. S4. (A) Osteosarcoma cell line xenografts were trea [file 13045_2021_1156_MOESM1_ESM.pdf]
